# The transcriptional landscape of endogenous retroelements delineates esophageal adenocarcinoma subtypes

**DOI:** 10.1093/narcan/zcad040

**Published:** 2023-07-26

**Authors:** Anastasiya Kazachenka, Jane Hc Loong, Jan Attig, George R Young, Piyali Ganguli, Ginny Devonshire, Nicola Grehan, The OCCAMS Consortium, Rebecca C Fitzgerald, Paul A W Edwards, Nicola Grehan, Barbara Nutzinger, Elwira Fidziukiewicz, Aisling M Redmond, Sujath Abbas, Adam Freeman, Elizabeth C Smyth, Maria O’Donovan, Ahmad Miremadi, Shalini Malhotra, Monika Tripathi, Calvin Cheah, Hannah Coles, Connor Flint, Matthew Eldridge, Maria Secrier, Ginny Devonshire, Sriganesh Jammula, Jim Davies, Charles Crichton, Nick Carroll, Richard H Hardwick, Peter Safranek, Andrew Hindmarsh, Vijayendran Sujendran, Stephen J Hayes, Yeng Ang, Andrew Sharrocks, Shaun R Preston, Izhar Bagwan, Vicki Save, Richard J E Skipworth, Ted R Hupp, J Robert O’Neill, Olga Tucker, Andrew Beggs, Philippe Taniere, Sonia Puig, Gianmarco Contino, Timothy J Underwood, Robert C Walker, Ben L Grace, Jesper Lagergren, James Gossage, Andrew Davies, Fuju Chang, Ula Mahadeva, Vicky Goh, Francesca D Ciccarelli, Grant Sanders, Richard Berrisford, David Chan, Ed Cheong, Bhaskar Kumar, L Sreedharan, Simon L Parsons, Irshad Soomro, Philip Kaye, John Saunders, Laurence Lovat, Rehan Haidry, Michael Scott, Sharmila Sothi, Suzy Lishman, George B Hanna, Christopher J Peters, Krishna Moorthy, Anna Grabowska, Richard Turkington, Damian McManus, Helen Coleman, Russell D Petty, Freddie Bartlett, Francesca D Ciccarelli, Rebecca C Fitzgerald, George Kassiotis

**Affiliations:** Retroviral Immunology Laboratory, The Francis Crick Institute, London, UK; Retroviral Immunology Laboratory, The Francis Crick Institute, London, UK; Retroviral Immunology Laboratory, The Francis Crick Institute, London, UK; Bioinformatics and Biostatistics Facility, The Francis Crick Institute, London, UK; Cancer Systems Biology Laboratory, The Francis Crick Institute, London, UK; School of Cancer and Pharmaceutical Sciences, King's College London, London, UK; Cancer Research UK Cambridge Institute, University of Cambridge, Cambridge, UK; Early Cancer Institute, Hutchison Research Centre, University of Cambridge, Cambridge, UK; Cancer Systems Biology Laboratory, The Francis Crick Institute, London, UK; School of Cancer and Pharmaceutical Sciences, King's College London, London, UK; Early Cancer Institute, Hutchison Research Centre, University of Cambridge, Cambridge, UK; Retroviral Immunology Laboratory, The Francis Crick Institute, London, UK; Department of Infectious Disease, Faculty of Medicine, Imperial College London, London, UK

## Abstract

Most cancer types exhibit aberrant transcriptional activity, including derepression of retrotransposable elements (RTEs). However, the degree, specificity and potential consequences of RTE transcriptional activation may differ substantially among cancer types and subtypes. Representing one extreme of the spectrum, we characterize the transcriptional activity of RTEs in cohorts of esophageal adenocarcinoma (EAC) and its precursor Barrett's esophagus (BE) from the OCCAMS (Oesophageal Cancer Clinical and Molecular Stratification) consortium, and from TCGA (The Cancer Genome Atlas). We found exceptionally high RTE inclusion in the EAC transcriptome, driven primarily by transcription of genes incorporating intronic or adjacent RTEs, rather than by autonomous RTE transcription. Nevertheless, numerous chimeric transcripts straddling RTEs and genes, and transcripts from stand-alone RTEs, particularly KLF5- and SOX9-controlled *HERVH* proviruses, were overexpressed specifically in EAC. Notably, incomplete mRNA splicing and EAC-characteristic intronic RTE inclusion was mirrored by relative loss of the respective fully-spliced, functional mRNA isoforms, consistent with compromised cellular fitness. Defective RNA splicing was linked with strong transcriptional activation of a *HERVH* provirus on Chr Xp22.32 and defined EAC subtypes with distinct molecular features and prognosis. Our study defines distinguishable RTE transcriptional profiles of EAC, reflecting distinct underlying processes and prognosis, thus providing a framework for targeted studies.

## Graphical Abstract

**Graphical Abstract:**
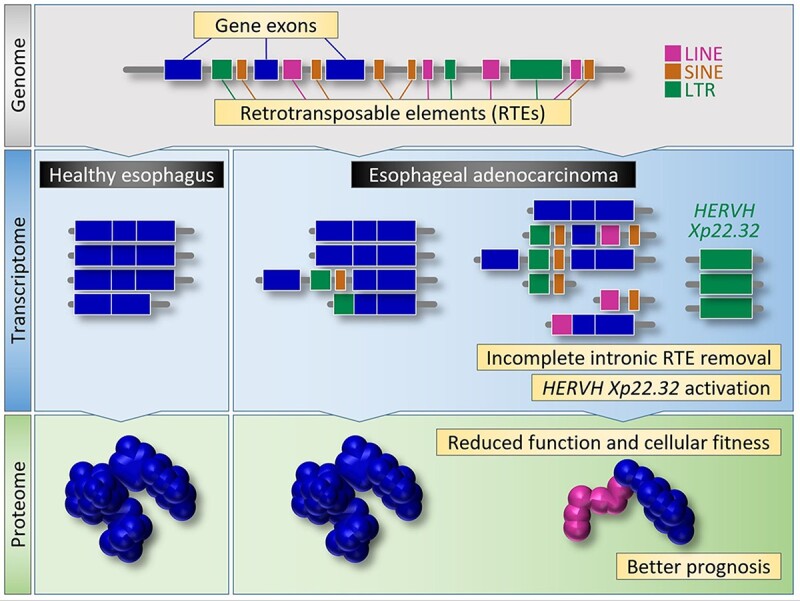

## INTRODUCTION

Gene expression depends on transcription, subsequent splicing and polyadenylation of nascent RNA, through recognition of specific motifs in genomic DNA. Between and within genes, however, reside numerous retrotransposable elements (RTEs) that can contribute transcription initiation signals, splice donor and acceptor sites, and polyadenylation signals, thereby contributing to or disrupting RNA production processes (1). The human genome harbors over 4 million RTE integrations of distinct phylogeny, genomic structure and replication life-cycle, with a vast majority of them being replication-defective due to accumulation of mutations and deletions (2,3). A major distinction is the presence of long-terminal repeats (LTRs) in human endogenous retroviruses (HERVs), originating from germline infection with exogenous retroviruses, and in mammalian apparent LTR retrotransposons (MaLRs). In contrast, long interspersed nuclear elements (LINEs) and short interspersed nuclear elements (SINEs), which include the expanded *Alu* elements, lack LTRs (2,3). Another pertinent distinction is the use of poly(T) by non-LTR LINE-1, SINE and the composite SINE-VNTR-*Alu* (SVA) elements, all of which rely on the LINE-1 replication machinery, for priming of reverse transcription (target-primed), which inserts poly(A) tails in DNA integration sites (2,3).

RTE expression has been found dysregulated, at least in part owing to epigenetic derepression, in most cancer types that have been examined, where it may have more pronounced effects on gene function and RNA production, as well as additional effects, such as induction of an interferon (IFN) response or creation of cancer-specific antigens (4,5). However, the degree or direction of RTE dysregulation is highly variable among different cancer types. Whilst average RTE transcription is reported upregulated in a majority of cancer types, it may also be downregulated in other types characterized by increased epigenetic repression, and individual RTE copies or families may also display opposing patters of dysregulation even in the same cancer type (4,6,7), highlighting cancer-specific and RTE-specific causal processes.

Using *de novo* transcriptome assembly, we have previously observed substantially increased aberrant transcription of LTR retroelements in esophageal carcinoma (ESCA), compared with healthy tissues or other cancer types, with ESCA being second only to testicular germ cell tumors (TGCT), where RTEs are dysregulated also as part of epigenetic reprogramming during spermatogenesis (8). ESCA is classified histologically and molecularly as squamous cell carcinoma (ESCC) or adenocarcinoma (EAC) (9), each associated with different risk factors. In particular, EAC is connected with Barrett's esophagus (BE), a condition of esophageal epithelium and an EAC precursor state (10). It was therefore unclear whether the pronounced LTR retroelement dysregulation in ESCA transcriptomes (8) tracks with EAC and its precursor BE, or whether it extends to all types of RTE, particularly the more numerous non-LTR type. Increased non-LTR retroelement activity in EAC is also suggested by observations that LINE-1 insertions are the most frequent type of somatic structural variation in EAC (11–14) and are also detected in pre-malignant BE (12,15).

In this work, we have utilised established TCGA cohorts, as well as a new extended cohort of EAC and BE from the OCCAMS consortium to define RTE transcriptional activity in EAC, and explore its origins and potential consequences. We identified a comprehensive list of RTE-overlapping transcripts overexpressed specifically in EAC, many of which were not previously annotated and from which we derived both diagnostic and prognostic RTE transcriptional signatures. We further pinpointed incomplete intron processing and intronic RTE removal, rather than autonomous ERE expression, as the origin of the exceptional RTE transcriptional diversity in EAC. In turn, we found that defective intron processing is associated with reduced cellular fitness, owing to reduced expression of the fully-spliced, functional mRNA isoforms. We further associated defective intronic RTE removal with strong transcriptional activation of distinct *HERVH* proviruses and of the *HERVH Xp22.32* provirus in particular, controlled by transcription factors KLF5 and SOX9. Most notably, we found that defective intronic RTE processing and associated *HERVH Xp22.32* activation defines a distinguishable subtype of EAC that exhibits more pronounced adenocarcinoma characteristics, is more divergent from its BE precursor and from ESCC, and is associated with reduced cancer cell fitness and, consequently better prognosis.

## MATERIALS AND METHODS

### OCCAMS (oesophageal cancer clinical and molecular stratification) cohorts

This work used previously published and newly collected samples from the OCCAMS study (REC. no. 10-H0305-1). OCCAMS is an observational study to determine the molecular drivers of EAC. Ethical approval was obtained from the Cambridgeshire 4 Research Ethics Committee, UK. Tissue was obtained with written, informed patient consent. All relevant ethical regulations were correctly followed and samples were fully anonymized. The OCCAMS whole genome sequencing (WGS) samples analyzed here included 99 previously described EAC samples (16) and an additional 128 EAC samples. Single nucleotide variants (SNVs), small indels and copy number alterations (CNAs) were called using Strelka v.1.0.13 (17) and ASCAT-NGS v.2.1 (18), as described previously (19). A total of 40 EAC cancer drivers frequently altered through SNVs or indels were derived from the Network of Cancer Genes database (NCG, http://www.network-cancer-genes.org) (20). Additionally, 34 drivers frequently altered through copy number alterations (CNA) were derived from the literature (9,16,19,21,22). Damaging alterations in these 74 EAC drivers were identified using ANNOVAR (April 2018) (23) and dbNSFP (24). Stopgain, stoploss and frameshift mutations were considered damaging. Missense and splicing mutations were further filtered to identify loss-of-function and gain-of-function alterations, as described previously (20). Additionally, drivers with copy number gains (CNA > 2 times sample ploidy), homozygous deletions (CNA = 0) and heterozygous deletion (CNA = 1) with a loss of function mutation in the other allele were considered to be damaged. The OCCAMS RNA sequencing (RNA-seq) samples (ribosomal RNA (rRNA)-depleted total RNA) analyzed here included 116 previously described EAC samples (16) and an additional 131 EAC and 110 BE samples. For RNA-seq raw data analyses, adapter and quality control trimming were carried out using Trimmomatic v0.36 (Bolger*et al*., 2014). Quality control of raw reads, carried out using FastQC, indicated the presence of bacterial RNA and residual rRNA reads in a majority of the samples. These reads were filtered out using BBsplit (BBMap v36.20) from BBTools suit (http://jgi.doe.gov/data-and-tools/bb-tools/) by aligning reads against the GRCh38/hg38 genome and the human ribosomal DNA complete repeating unit (GenBank: U13369.1). Samples that ended up having less than 10^6^ paired reads after removal of bacterial RNA and rRNA reads were excluded from downstream analyses.

### Transcript identification, read mapping and quantitation

OCCAMS RNA-seq samples that passed quality control were to the GRCh38/hg38 human genome using HISAT2 v2.1.0 (25). Additional RNA-seq samples were downloaded from TCGA (poly(A) selected RNA) as .bam files and converted to .fasta files using SAMtools v1.8 (26). Downstream analysis of TCGA samples and other publicly available RNA-seq datasets used in this study was carried out as for OCCAMS cohorts excluding bacterial RNA and rRNA read filtering step. Although poly(A) RNA selection may underestimate transcripts from satellite repeats, transcripts from RTEs were previously found similarly represented in total and poly(A)-selected RNA (27). Annotated gene and repeat expression were calculated by featureCounts (part of the Subread package v1.5.0) (28) using GENCODE.v29 basic (29) and a custom repeat annotation (30). Genic repeats were defined as those with at least one nucleotide overlap with annotated gene bodies, with the rest of the repeats defined as intergenic. To prevent ambiguity, only reads that could be uniquely assigned to a single feature were counted. Long-read RNA-seq samples were aligned to the GRCh38/hg38 human genome using minimap2 v2.17 (31). For assemblies of long-read RNA-seq reads, the obtained .bam files were first converted to bed12 using bam2bed12.py script from FLAIR suit (32). High-confidence isoforms were selected using ‘collapse’ function from flair.py script (32). ChIP-seq datasets were trimmed using Trimmomatic v0.36 (33) and aligned to the GRCh38/hg38 human genome using Bowtie 2 v2.2.9 (34). Additional transcripts were previously *de novo* assembled on a subset of the RNA-seq data from TCGA (8). Samples from TCGA were downloaded through the *gdc-client* application and the *.bam* files were parsed with a custom Bash pipeline using GNU parallel (35). RNA-seq data from TCGA, GTEx, CCLE OCCAMS and listed previous studies were mapped to our *de novo* cancer transcriptome assembly and counted as previously described (8). Briefly, transcripts per million (TPM) values were calculated for all transcripts in the transcript assembly (8) with a custom Bash pipeline and Salmon v0.8.2 (36), which uses a probabilistic model for assigning reads aligning to multiple transcript isoforms, based on the abundance of reads unique to each isoform (36). The 4844 ESCA-overexpressed transcripts were selected based on median expression in ESCA >0.5 TPM, with the 90th percentile of expression in the respective healthy tissues or the maximum median expression in any healthy tissue at least 3× lower than the 75th percentile of expression in ESCA, and <0.5 TPM. We separately quantified expression of annotated genes by using a transcript index with all GENCODE transcript_support_level:1 entries and collapsing counts for the same gene. For quantitation of exon versus intron representation, the same pipeline was followed, except counts were collapsed for all annotated exons and all introns for the same gene, separately. Read count tables were additionally imported into Qlucore Omics Explorer v3.8 (Qlucore, Lund, Sweden) for further downstream expression analyses and visualization. Splice junctions were visualized using the Integrative Genome Viewer (IGV) v2.4.19 (37).

### Repeat annotation

Repeat regions were annotated as previously described (30). Briefly, hidden Markov models (HMMs) representing known Human repeat families (Dfam 2.0 library v150923) were used to annotate GRCh38 using RepeatMasker, configured with nhmmer. RepeatMasker annotates LTR and internal regions separately, thus tabular outputs were parsed to merge adjacent annotations for the same element.

### Cellular deconvolution of bulk RNA-seq data

Frequencies of immune cell populations in patient samples were estimated by cellular deconvolution of bulk RNA-seq data using the CIBERSORTx method (https://cibersortx.stanford.edu) (38).

### Consensus motif identification

Consensus motifs were identified at the 5′ and 3′ ends of all fully intronic transcripts by sequence alignments of the terminal 40 bp at either end using the WebLogo tool (https://weblogo.berkeley.edu/logo.cgi) (39). The results were plotted as sequence logos.

### Functional gene annotation by gene ontology

Pathway analyses were performed using g:Profiler (https://biit.cs.ut.ee/gprofiler) with genes ordered by the degree of differential expression. P values were estimated by hypergeometric distribution tests and adjusted by multiple testing correction using the g:SCS (set counts and sizes) algorithm, integral to the g:Profiler server (40).

### Survival analysis and hazard ratio calculations

For survival analysis, all OCCAMS EAC samples with survival data recorded were used. To test if expression of a transcript of interest correlated with patients’ survival, we identified the patients in the bottom and top percentile expression (‘low’ versus ‘high’ expression). Survival analysis was done using the survfit function of the survival R package (v2.42), using overall survival time. To compare curves between low and high expression tertiles, log-rank testing was used and a Cox regression model was built to test the assumption of proportional hazards holds. Hazard odd ratios are given based on the Cox regression model. Similarly, a Cox regression model was used to compare survival between multiple expression clusters.

### Cell lines

OE19 (RRID: CVCL_1622), HARA (RRID: CVCL_2914) and LK-2 cells (RRID: CVCL_1377) were obtained from the Cell Services facility of The Francis Crick Institute and verified as mycoplasma-free. All human cell lines were further validated by DNA fingerprinting. Both human lung squamous cell carcinoma cell lines - HARA and LK2 were grown in RPMI 1640 medium (Gibco) with 10% heat-inactivated fetal bovine serum (Gibco), 2 mM l-glutamine (Thermo Fisher Scientific), 10 μM 2-mercaptoethanol (Sigma-Aldrich), 100 μM non-essential amino acids (Sigma-Aldrich) penicillin (100 U/ml) (Thermo Fisher Scientific), and streptomycin (0.1 mg/ml) (Thermo Fisher Scientific). Esophageal adenocarcinoma cell line - OE19 were grown in RPMI 1640 medium (Gibco) with 10% heat inactivated fetal bovine serum (Gibco), 2 mM l-glutamine (Thermo Fisher Scientific), penicillin (100 U/ml) (Thermo Fisher Scientific), and streptomycin (0.1 mg/ml) (Thermo Fisher Scientific).

### Cell transfections

OE19 cells were seeded at a density of 300 000 cells/well in 2 ml of culture media 24 hours prior transfection in 6-well plates. Cells were then transfected with 5 μg of plasmid each expressing the following transcription factors: KLF5 (pcDNA3.1-KLF5, Genewiz) or SOX9 (pcDNA3.1-SOX9, Genewiz) using Lipofectamine 3000 transfection reagent (Thermo Fisher). RNA was extracted 48 hours after transfection.

### Reverse transcriptase-based quantitative PCR (RT-qPCR)

RNA was extracted using the RNeasy kit (Qiagen). cDNA was synthesized using the Maxima First Strand cDNA Synthesis Kit (Thermo Fisher), and qPCR performed using Applied Biosystems Fast SYBR Green (Thermo Fisher) using the following primers:

Target Forward Reverse

*HERVH Xp22.32*GGCAGCGACTCCCAGAGA TGATGGTCTACAGGGCTTCC

*HERVH-CALB1*AGCCCAAGAAACATCTCACCAA CAGCCTTCTTTCGCGCCTG

*HPRT*TGACACTGGCAAAACAATGCA GGTCCTTTTCACCAGCAAGCT

Values were normalised to HPRT expression using the ΔC_T_ method.

### RT-PCR and amplicon sanger sequencing

cDNA from HARA and LK-2 cells was used as template for PCR amplification, performed using KOD Hot Start Master Mix (Sigma) with the following primers:

Target Forward Reverse

*L1PA2-L1PB1*TTTGACTCAGAAAGGGAACT GTACGCCAATTTTAATTGTT

Separately, cDNA from LK-2 cells was amplified using nested PCR with the following primers:

Target Forward Reverse

*L1PA2-L1PB1* first round TTTGACTCAGAAAGGGAACT AGGTAGTGGGATGCCTCCAG

*L1PA2-L1PB1* second round GCAATGCCTCACCCTGCTTC GGTCTTGCACCTCCTTGGTT

The PCR products were Sanger sequenced by Genewiz, Essex, UK, using the same primers.

### Statistical analyses

Statistical comparisons were made using GraphPad Prism 7 (GraphPad Software), SigmaPlot 14.0., or R (versions 3.6.1–4.0.0). Parametric comparisons of normally distributed values that satisfied the variance criteria were made by unpaired or paired Student's *t*-tests or One Way Analysis of variance (ANOVA) tests with Bonferroni correction for multiple comparisons. Data that did not pass the variance test were compared with non-parametric two-tailed Mann–Whitney Rank Sum tests (for unpaired comparisons), Wilcoxon Signed Rank test (for paired comparisons) or ANOVA on Ranks tests with Tukey or Dunn correction for multiple comparisons. Multi-region data were compared using a linear mixed effects model with each patient as a random effect.

## RESULTS

### Increased RTE inclusion in esophageal and stomach cancer transcriptomes

**Figure 1.**
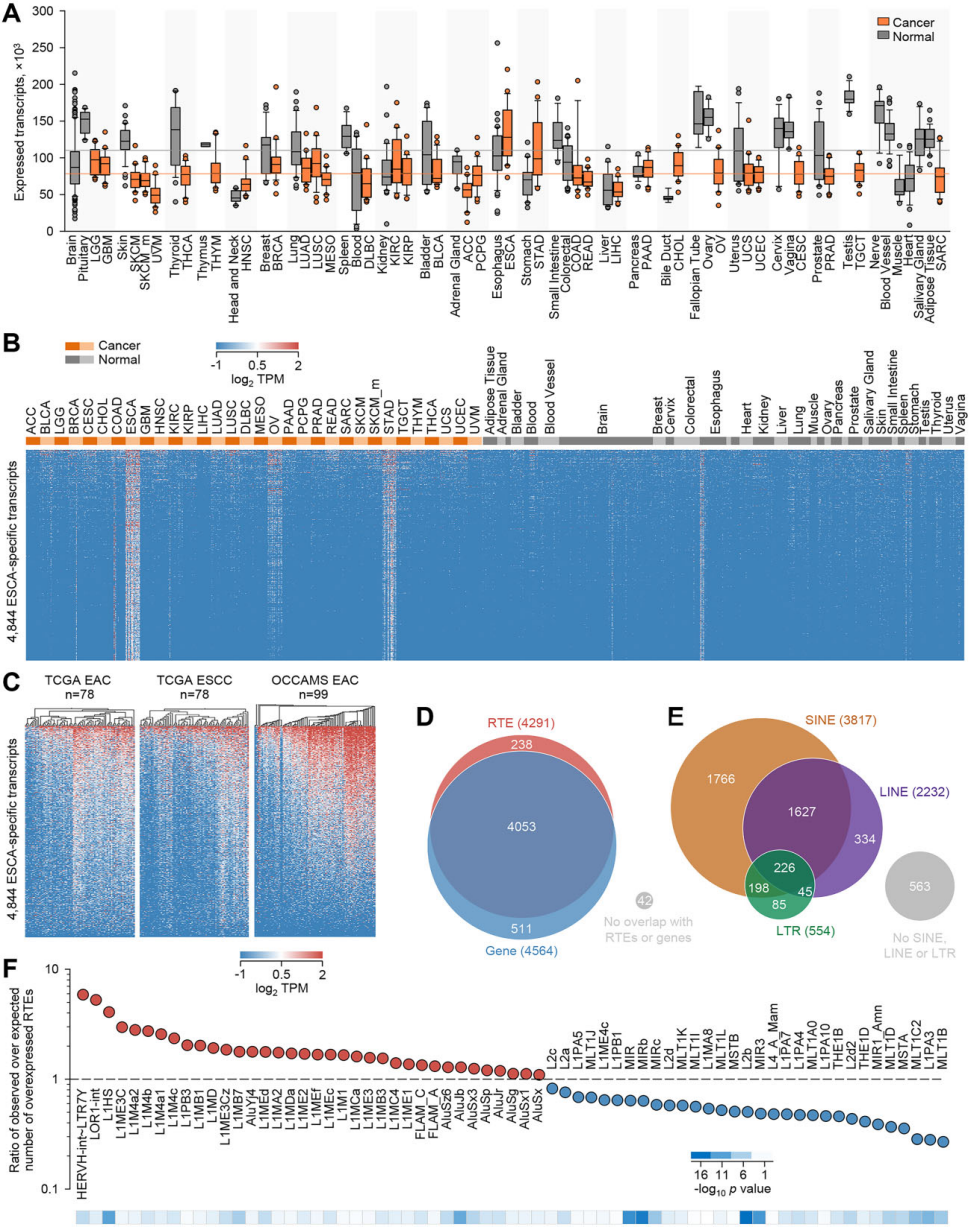
Increased inclusion of RTEs in the ESCA and STAD transcriptomes. (**A**) Number of transcripts expressed (≥0.5 TPM) in the indicated cancer (*n* = 24 per cancer type) or normal tissue samples (*n* = 2–156 per tissue type). Box plots denote median value and quartiles, whiskers denote 1.5× the interquartile range, and individual points denote outliers. (**B**) Heatmap of expression of 4844 ESCA-overexpressed transcripts in the same samples as in (A). (**C**) Heatmap of expression of 4844 ESCA-overexpressed transcripts in extended TCGA EAC and ESCC cohorts and in an additional OCCAMS EAC cohort. (D, E) Overlap of the 4844 ESCA-overexpressed transcripts with RTEs or annotated genes (**D**) and according to the RTE group (**E**). (**F**) Enrichment of the indicated RTE subfamily in the 4844 ESCA-overexpressed transcripts, compared with all assembled transcripts (*P* values were calculated with Fisher's exact tests).

To examine if the increased inclusion of LTR elements previously seen in ESCA transcriptomes (8) extended beyond these elements, we compared measures of overall transcriptome complexity across different cancer types and respective healthy tissues. We considered the total number of assembled transcripts expressed at ≥0.5 TPM in each sample as an indirect measure of transcriptome complexity. The number of expressed transcripts overlapping an LTR element was proportional to the total, with approximately 14% of all transcripts including an LTR element in both malignant and healthy tissues (Supplementary Figure S1A). A far greater proportion of transcripts included a non-LTR RTE (77% and 82% in healthy and malignant tissues, respectively) than an LTR RTE (Supplementary Figure S1B). According to this measure, healthy tissues varied substantially in transcriptome complexity, as expected, given distinct cellular composition and differentiation (Figure 1A), independently of sequencing depth (Supplementary Figure S1C). Cancer samples exhibited overall lower complexity and, in most cases, lower than the respective healthy samples, with the exception of ESCA and stomach adenocarcinoma (STAD), where transcriptome complexity was significantly increased (*P* = 0.005 and *P* < 0.001, respectively, Mann–Whitney Rank Sum test) (Figure 1A). Thus, increased activity of LTR elements in ESCA (8) appeared to reflect increased overall transcriptome diversity.

We next selected 4844 assembled contigs recurrently present in both ESCA and STAD, but minimally in healthy tissues for further analysis. Estimated expression of these was shared also with ovarian serous cystadenocarcinoma (OV) and, to a lesser degree, colon adenocarcinoma (COAD) (Figure 1B). Expression of the selected transcripts was not observed in non-malignant tissue samples from TCGA or GTEx, with the exception of three particular esophagus samples from TCGA (Figure 1B). The latter, however, were all tumor-adjacent samples, rather than completely healthy tissue, which may have altered their transcriptional profile (41).

The vast majority (98.9%) of the selected transcripts were also found expressed in two extended cohorts of esophageal adenocarcinoma (EAC, *n* = 78) and esophageal squamous cell carcinoma (ESCC, *n* = 78) from TCGA, as well as an additional cohort of EAC (*n* = 99) from OCCAMS (16) (Figure 1C). Moreover, the assembled transcripts appeared co-expressed in individual samples (Figure 1C), implying they were generated by a common mechanism.

A small number of the selected transcripts (238) comprised only RTEs and an even smaller number (42) did not overlap with any annotated region (Figure 1D, Supplementary Table S1). The remaining transcripts (4564) partially overlapped with 2144 annotated genes (an average of 2.1 transcripts per gene), and a vast majority of these (4053) also overlapped with RTEs (Figure 1D). A great majority of the transcripts (4281) included one or more elements from the three major groups, with SINEs being by far the most frequent, followed by LINEs, and LTR elements the least frequent (Figure 1E). Compared with all assembled transcripts, the overexpressed 4844 transcripts were significantly enriched for L1 LINE subfamilies, including *L1HS*, and certain SINE subfamilies, particularly *Alu* subfamilies (Figure 1F). LTR elements were relatively absent, with the notable exception of the *HERVH* subfamily, which was the most enriched in the selected transcripts (Figure 1F; Supplementary Table S1). In contrast, the evolutionary older MIR SINE and L2 LINE subfamilies appeared overall underrepresented in the selected transcripts (Figure 1F).

For orthogonal validation of these findings, RTE expression was separately quantified using featureCounts and a custom repeat annotation (30), excluding multi-mapping reads, in the TCGA EAC cohort (Supplementary Figure S2). This analysis identified 1179 RTEs as significantly differentially expressed (>6-fold-change, *P* < 0.05, *q* < 0.05) between EAC and normal esophagus TCGA samples, a great majority of which (984) were genic (Supplementary Figure S2A). They included comparable proportions of LINEs and SINEs and a smaller proportion of LTR elements (Supplementary Figure S2B). In agreement with our transcriptome-based quantitation, enrichment analysis of read counts identified L1 LINE subfamilies, including *L1HS*, and the *HERVH* subfamily of LTR elements as significantly enriched (Supplementary Figure S2C). However, the overexpressed *Alu* subfamilies appeared underrepresented, likely due to an underestimation of their actual expression by the discarding of multi-mapping reads, which would affect multi-copy subfamilies disproportionally (Supplementary Figure S2C). Indeed, with featureCounts, we observed a strong effect of the number of copies of a given RTE in the total transcriptome on its relative enrichment in the EAC-overexpressed RTEs (Supplementary Figure S2D).

Together, these findings suggested that the increased transcriptional representation of RTEs in esophageal and stomach cancer transcriptomes resulted primarily from gene transcription incorporating intronic or adjacent RTEs, rather than of autonomous transcription of stand-alone, intergenic RTEs.

### Aberrant RNA splicing of RTEs in the esophageal cancer transcriptome

**Figure 2.**
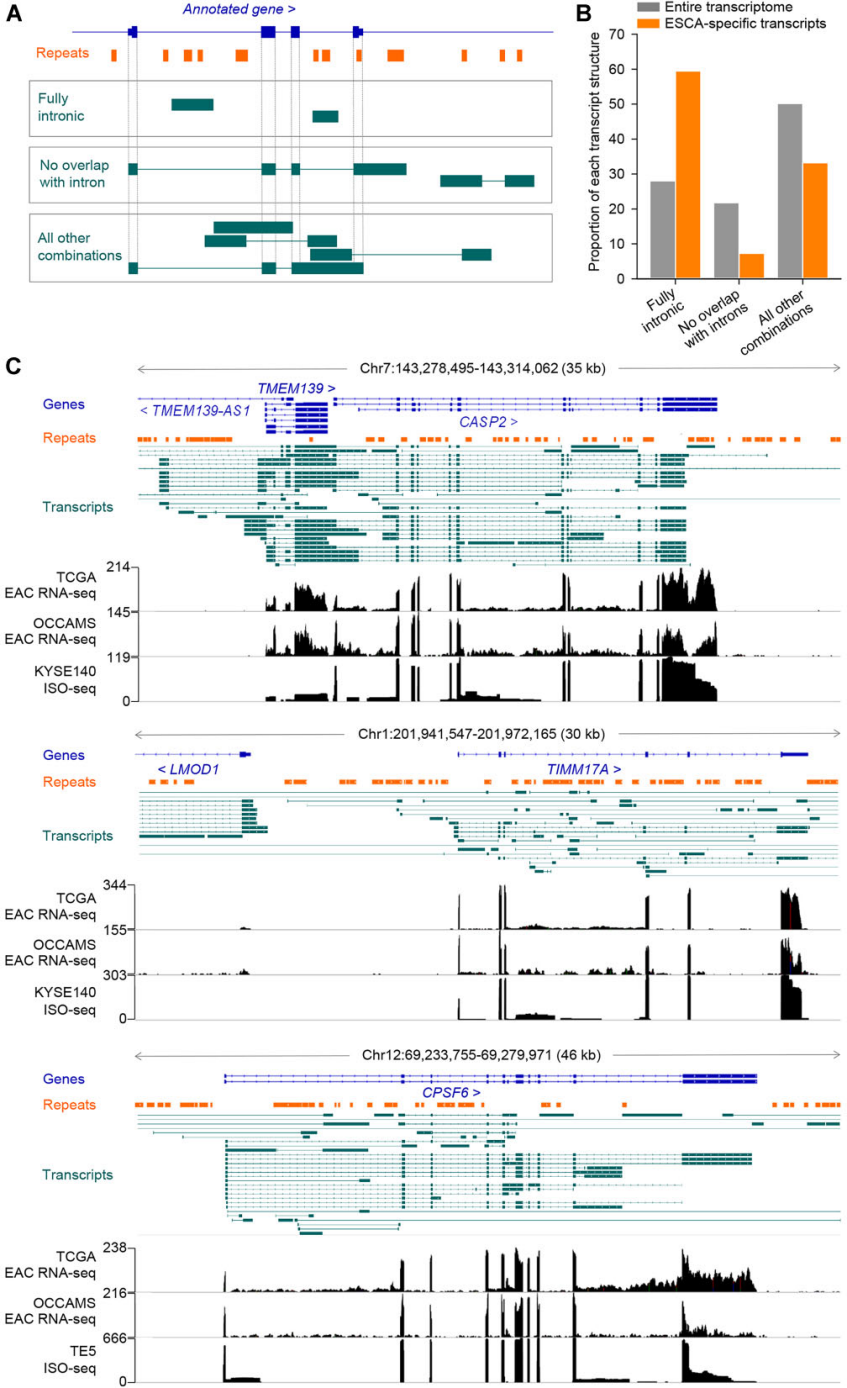
Aberrant RNA splicing in the EAC transcriptome. (**A**) Schematic representation of the classification of assembled transcripts according to their location relative to the nearest gene body. (**B**) Proportion of the indicated class of transcript in ESCA-specific and in all assembled transcripts. (**C**) GENCODE annotated transcripts (Genes), RTEs (Repeats), assembled transcripts, RNA-seq traces of representative TCGA EAC and OCCAMS EAC samples, and ISO-seq traces of the ESCC cell lines KYSE140 and TE5 (PRJNA515570), at the *CASP2*, *TIMM17A* and *CPSF6* loci.

To investigate a potential mechanism underlying the preferential inclusion of genic, rather than intergenic RTEs, we separated ESCA-overexpressed transcripts into those that were entirely within annotated introns, those that did not include any intronic sequences and all other combinations (Figure 2A). Compared with the entire transcriptome, the ESCA-overexpressed transcripts were enriched for fully intronic contigs, at the expense of those not overlapping with introns (Figure 2B). Nevertheless, a third of the ESCA-overexpressed transcripts comprised combinations of exonic and intronic or intergenic RTEs (Figure 2B).

The abundant seemingly fully intronic reads were not a result of DNA contamination as they covered intronic but not intergenic RTEs, as exemplified in the *CASP2*, *TIMM17A* or *CPSF6* loci, where RNA-seq reads mapped to numerous intronic but not intergenic RTEs (Figure 2C). Moreover, they were independent from RNA selection methods as they were detected both in TCGA and in OCCAMS RNA-seq data, which were generated using poly(A) selected RNA and rRNA-depleted total RNA, respectively (Figure 2C). Lastly, fully intronic, but not intergenic contigs spanning RTEs were also detected in long-read ISO-seq data from esophageal cancer cell lines (42), coinciding with TCGA and in OCCAMS RNA-seq peaks (Figure 2C). These data indicated incomplete RNA splicing in ESCA affecting multiple introns of a given gene, rather than retention of specific introns (Figure 2C), and was therefore likely distinct from intron retention.

Fully intronic contigs detected in ISO-seq data exhibited more defined boundaries than mapping of shorter RNA-seq reads and this offered the opportunity to examine the presence of repeats at either end of the contig. We noted that fully intronic contigs appeared to be initiated or terminated typically at an RTE (Supplementary Figure S3A, B) and we reasoned that this could arise from priming of reverse transcription during the cDNA synthesis step of library preparation at poly(A) tails in such RTEs, as has been suggested for intronic reads identified in single-cell (sc) RNA-seq data (43). Indeed, fully intronic contigs in ISO-seq libraries had poly(A) or poly(U) tracts at either end, depending on orientation relative to the gene, and were enriched for *Alu* SINEs, particularly of the most recent members *AluJb* and *AluSx* (Supplementary Figure S3C, D).

In addition to the generation of seemingly fully intronic contigs, large introns also exhibited evidence for splicing between flanking exons and intronic RTEs, likely resulting from incomplete recursive splicing (Supplementary Figure S4). Splicing between gene exons and intronic RTEs involved canonical and non-canonical donor and acceptor splice sites often in close proximity in the same intronic RTE (Supplementary Figure S4). Novel splicing was also detected between exonic RTEs, usually inverted *Alu* repeats in 3′ UTRs of annotated genes. In many cases, such apparent splicing was an artifact created during library preparation, where reverse transcription omits hairpin structures created by inverted *Alu* repeats, as previously described (44). However, actual splicing events were supported for inverted *Alu* repeats in 3′ UTRs of certain genes, such as *METTL16* and *MRPL30* (Supplementary Figure S5). In these cases, splicing involved canonical splice donor and acceptor sites and spliced reads spanning ADAR-edited inverted *Alu* repeats were detected in direct long-read RNA-seq data from HEK293T cells (Supplementary Figure S5), which is considered free from such artifacts (44).

Other types of chimeric transcripts included fully or partially annotated and unannotated isoforms transcribed from known genes and representing fully spliced, mature mRNAs that included RTEs as alternative exons or alternative promoters, as exemplified by the *CASP8*, *EPB41L5* or *DNAJC5* loci (Supplementary Figure S6-S8).

Therefore, autonomous transcription of stand-alone RTEs was a relatively minor contributor to the increased RTE representation in ESCA transcriptomes, which was instead caused primarily by transcription of RTEs within annotated genes and inclusion in alternatively spliced isoforms of annotated genes or transcripts overlapping with annotated gene bodies.

### Diagnostic and prognostic properties of RTE transcriptional inclusion in EAC

**Figure 3.**
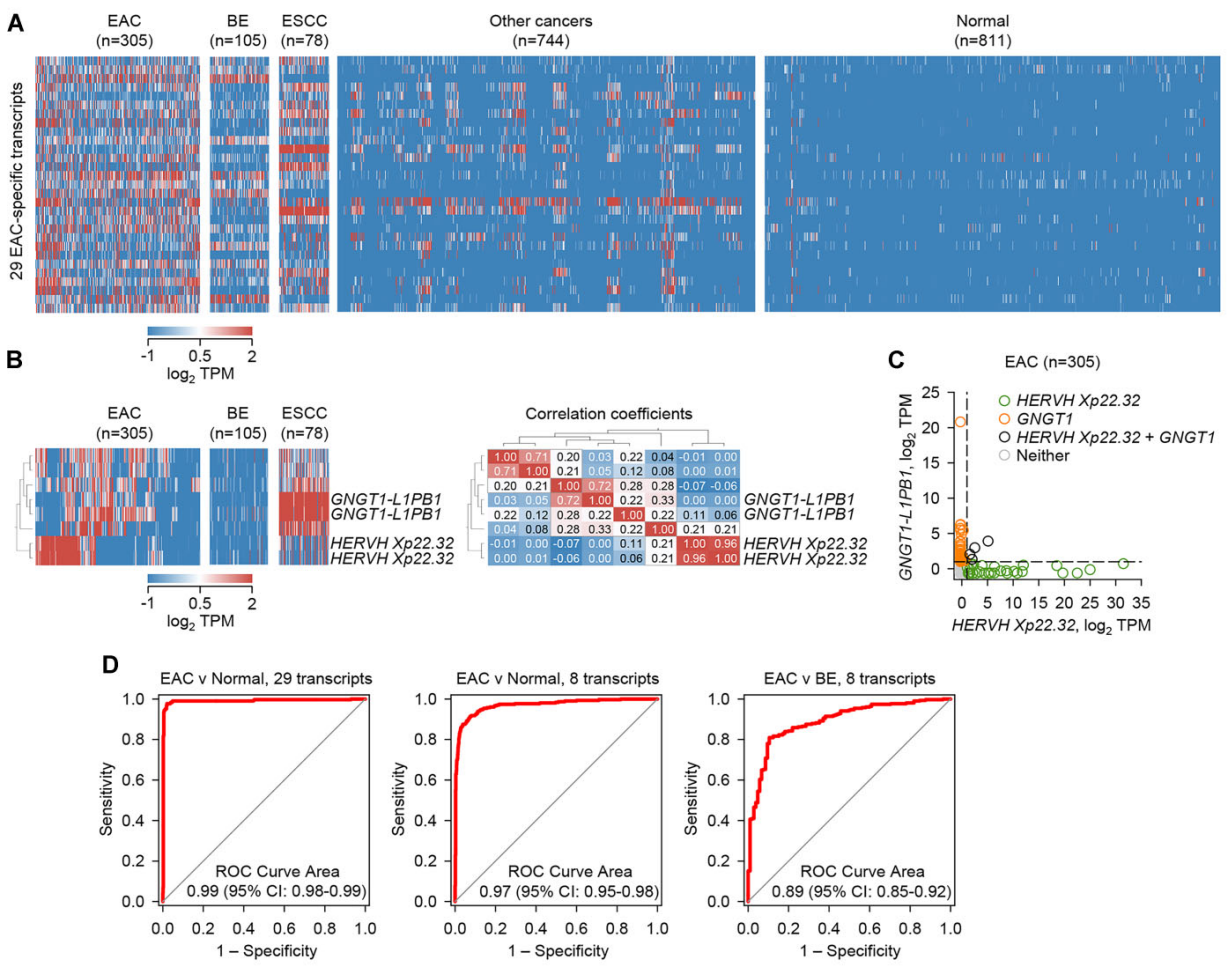
Diagnostic properties of RTE transcriptional inclusion in EAC. (**A**) Heatmap of expression of 29 EAC-overexpressed diagnostic transcripts in pooled TCGA and OCCAMS EAC samples, OCCAMS BE samples, TCGA ESCC samples, TCGA samples representing 30 other cancer types and pooled TCGA and GTEx normal tissue samples. (**B**) Heatmap of expression of 8 EAC-overexpressed transcripts that distinguish EAC and BE in TCGA and OCCAMS EAC samples, OCCAMS BE samples and TCGA ESCC samples (left) and correlation coefficients of the expression of these 8 transcripts in EAC samples (right). (**C**) Correlation of *HERVH Xp22.32* and *GNGT1-L1PB1* expression (sum TPMs of the two transcripts from each locus) in TCGA and OCCAMS EAC samples. (**D**) Receiver operating characteristic (ROC) curves of the performance of the sum of the *z*-scores of the 29 or 8 diagnostic transcripts in the indicated comparison of pooled TCGA and OCCAMS EAC samples, OCCAMS BE samples, and pooled TCGA and GTEx normal tissue samples.

We examined if the predictable inclusion of RTEs in EAC-specific RNA transcripts could improve EAC detection and diagnosis. To this end, we defined a signature of 29 transcripts (Supplementary Table S2) from the previously selected 4844 EAC-specific transcripts recurrently expressed in TCGA EAC (n = 78) and OCCAMS EAC (*n* = 99) publicly available samples, as well as an additional OCCAMS EAC cohort (*n* = 128) (Figure 3A). Many of these 29 transcripts were also expressed in BE samples, as well as in ESCC and other cancer indications, but were absent from any normal tissue we analyzed (Figure 3A). Moreover, 8 of the selected 29 transcripts were highly specific to EAC when compared with BE samples, in which they were not expressed (Figure 3B). These included two transcripts from the *GNGT1* locus exonising an L1 element (*GNGT1-L1PB1*), which was highly expressed also in ESCC, and two from a stand-alone *HERVH* provirus on Chr Xp22.32, which were relatively absent from ESCC (Figure 3B). Notably, expression of these 8 transcripts was largely non-overlapping in EAC, with *GNGT1-L1PB1* and *HERVH Xp22.32* expressed in a mutually exclusive manner (Figure 3B, C), suggesting that they represented distinct EAC subtypes.

Whereas *HERVH Xp22*.32 transcripts corresponded to an annotated *HERVH* provirus that has been previously reported highly upregulated in COAD (45), the *GNGT1-L1PB1* transcripts were not previously annotated. Inspection of the locus revealed that they were partially assembled transcripts belonging to a larger transcript, which originated at an *L1PA2* element >320 kb upstream of the *GNGT1* gene (Supplementary Figure S9A). This transcription start site and first exon matched the annotated *GNGT1-205* isoform (ENST00000455502.5). A transcript matching *GNGT1-205* was independently reported in a recent pan-cancer analysis (referred to there as L1PA2_GNGT1), where it was also found to produce antigenic peptides from an alternative open reading frame, largely embedded in the *L1PA2* element (46). However, splicing was found here considerably more frequently between the common initiating *L1PA2* element and a second *L1PB1* element, where transcription terminated without extending to the remaining *GNGT1* gene (Supplementary Figure S9A). The latter transcript (referred to here as *L1PA2-L1PB1*), was also detected in ISO-seq data from the ESCC cell line KYSE140 (Supplementary Figure S9A) and canonical donor and acceptor splice sites were confirmed by sequencing of RT-PCR amplicons from HARA and LK-2 cells (Supplementary Figure S9B). *L1PA2-L1PB1* expression was significantly upregulated in multiple types of cancer and was found at higher levels than transcription of *GNGT1*, which was also cancer-specific (Supplementary Figure S9C).

To evaluate the diagnostic properties of the EAC-specific transcripts, we calculated the cumulative expression of the eight selected transcripts (by taking the sum of the *z*-scores of all transcripts in all available samples). When applied to the 29 selected transcripts, this metric distinguished EAC and normal samples with 99% sensitivity and specificity (Figure 3D). Restricting the analysis to the 8 selected transcripts largely retained the ability to separate EAC and normal samples (97%) and additionally separated EAC and BE samples with reasonable sensitivity and specificity (89%) (Figure 3D). These results highlighted characteristic transcriptional changes in EAC that can be revealed by analysis of only a few selected loci.

**Figure 4.**
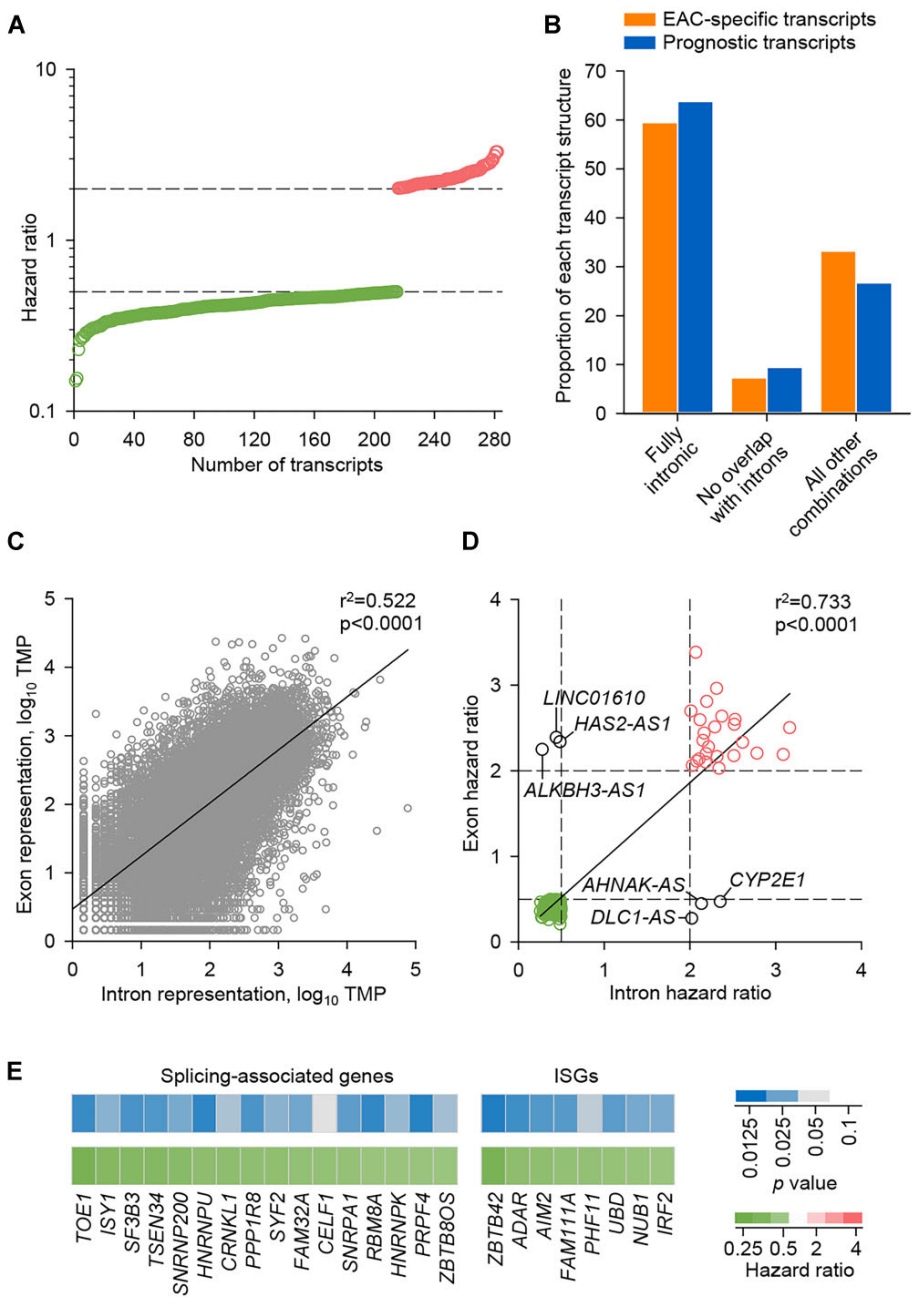
Prognostic properties of RTE transcriptional inclusion in EAC. (**A**) Mean hazard ratios for the 282 of the EAC-specific transcripts that exhibited a significant correlation with EAC survival (*P*< 0.05 in both OCCAMS EAC cohorts separately; hazard ratio ≥2 or ≤0.5). (**B**) Proportion of the indicated class of transcript in the 282 prognostic and in all ESCA-specific transcripts. (**C**) Correlation between exon and intron representation in the EAC transcriptome. Symbols represent individual genes in a representative OCCAMS EAC sample. (**D**) Correlation of the mean hazard ratios for 204 EAC-specific genes where both exon and intron expression correlated significantly with EAC survival when considered separately (*P*< 0.05 in both OCCAMS EAC cohorts separately; hazard ratio ≥2 or ≤0.5). (**E**) Heatmaps of mean p values and mean hazard ratios for prognostic splicing-associated genes and ISGs.

We next investigated if distinct EAC subtypes indicated by non-overlapping expression of some of the diagnostic EAC-specific transcripts may also follow different disease trajectories. To explore this possibility, we estimated the potential effect of aberrant RTE transcriptional inclusion on EAC survival, calculated as the hazard ratio for each OCCAMS EAC cohort separately, for additional validation. Of the 4593 EAC-specific transcripts expressed in both cohorts, 282 were significantly prognostic (*P* < 0.05 in both cohorts separately; hazard ratio ≥2 or ≤0.5) (Supplementary Table S3), with a majority (215) being protective (hazard ratio ≤ 0.5) (Figure 4A). A majority of prognostic transcripts were fully intronic (Figure 4B), as would be expected for any fraction of the EAC-specific transcripts.

As fully intronic transcripts often resulted from incomplete intron splicing in EAC, we examined if their association with survival reflected the expression of the genes in which they resided. As an independent measure of incomplete intron splicing indicated by the contigs in our assembly, we additionally calculated the expression of each gene considering exons and introns separately (Materials and Methods). Given that introns only from transcribed genes can be present in the transcriptome, we observed a significant positive correlation between exon and intron representation for each gene, but also considerable variation between genes (Figure 4C). Hazard ratio calculations identified 410 and 364 prognostic genes, when exon and intron expression were considered separately, respectively, with 204 of these at the intersection. For the majority of the intersecting 204 genes, exon and intron expression correlated with survival in the same direction, with a majority again being protective (hazard ratio ≤ 0.5), with the exception of six genes, whose intron expression was associated significantly with survival but in the opposite direction than that of exon expression (Figure 4D). However, all six of these represented shared introns between the gene of interest and overlapping antisense transcripts (Figure 4D).

These findings indicated that the survival association of increased intron representation in EAC reflected a contribution of individual genes, in which these introns resided, as well as the underlying process responsible for their incomplete removal. Indeed, the genes associated with longer EAC survival comprised several splicing factors, including *CRNKL1* and *HNRNPU*, which have been previously associated with EAC survival (47), and interferon signature genes (ISGs) (Figure 4E).

### *HERVH* transcriptional activation correlates with better EAC prognosis

**Figure 5.**
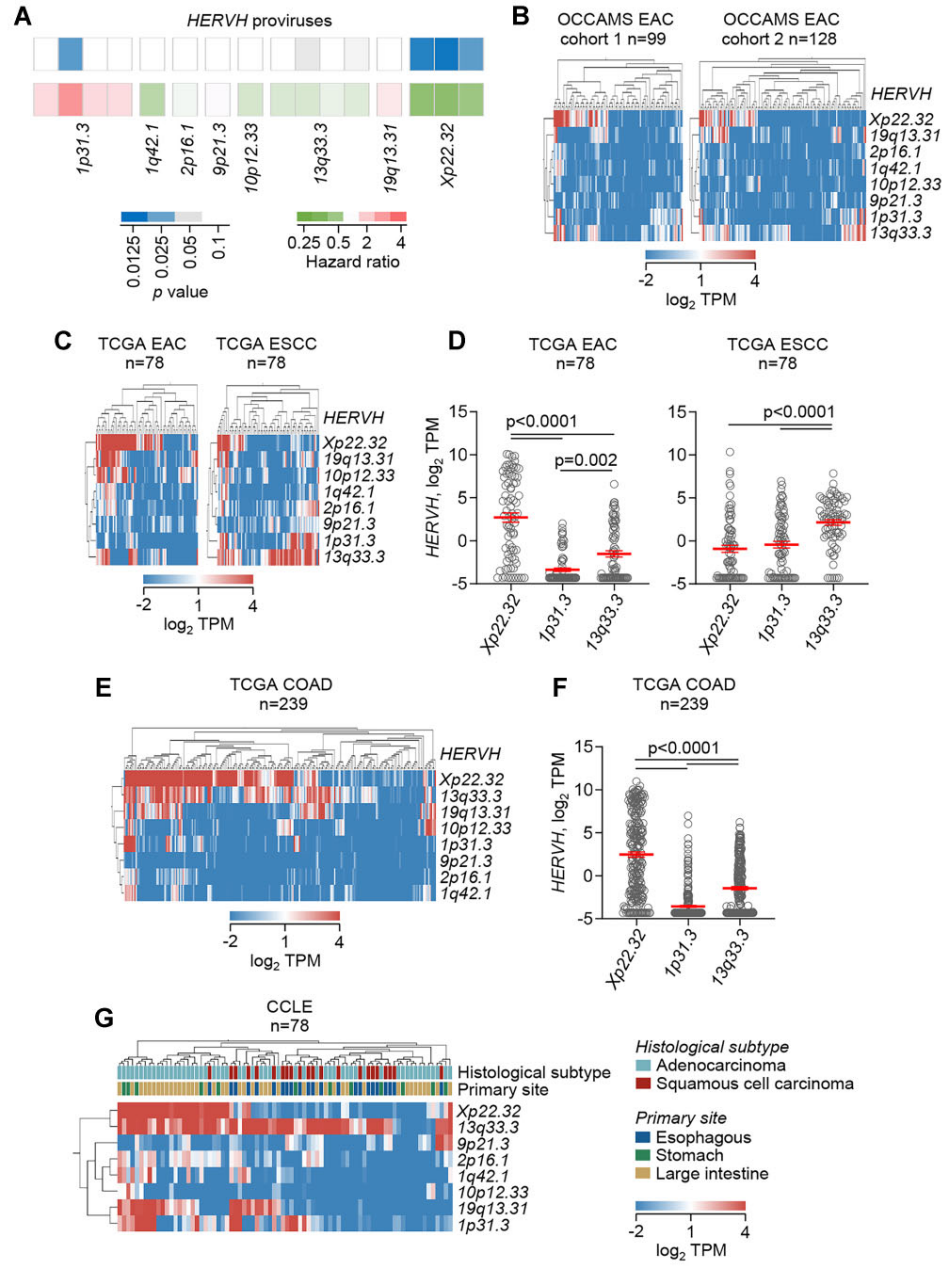
Pattern of *HERVH* expression in esophageal and colon cancers. (**A**) Heatmaps of mean *P* values and mean hazard ratios for the indicated *HERVH* proviruses calculated for survival of each OCCAMS EAC cohorts separately. (**B**) Heatmaps of expression of the indicated *HERVH* proviruses in hierarchically clustered samples from the two OCCAMS EAC cohorts. (**C**) Heatmaps of expression of the indicated *HERVH* proviruses in hierarchically clustered samples from TCGA EAC and TCGA ESCC. (**D**) Mean (±SEM) expression of *HERVH Xp22*.32, *HERVH 1p31.3* and *HERVH 13q33.3* proviruses in TCGA EAC and TCGA ESCC samples. (**E**) Heatmap of expression of the indicated *HERVH* proviruses in hierarchically clustered samples from TCGA COAD. (**F**) Mean (±SEM) expression of *HERVH Xp22*.32, *HERVH 1p31.3* and *HERVH 13q33.3* proviruses in TCGA COAD samples. (**G**) Heatmap of expression of the indicated *HERVH* proviruses in hierarchically clustered samples from CCLE cell lines derived from the esophagus, stomach or large intestine.

Whereas a majority of prognostic EAC-specific transcripts were intronic contigs associated with gene transcription and aberrant splicing, few corresponded to stand-alone RTEs (Supplementary Table S3). The latter included the diagnostic *HERVH Xp22.32* provirus and another *HERVH* provirus on Chr 1p31.3, significantly associated with better and worse EAC prognosis, respectively (Supplementary Table S3; Figure 5A). Although the overexpression of *HERVH Xp22*.32 in COAD has long been reported (45,48), its significance remained uncertain. Global *HERVH* upregulation has very recently been linked with worse COAD survival, but this related predominantly to *HERVH 1p31.3* and a separate *HERVH* provirus on Chr 6q24.2, whereas *HERVH Xp22*.32 was not reported to affect survival (49). Consistent with this recent report, we found that *HERVH 1p31.3* activation associated with worse EAC survival, in contrast to *HERVH Xp22*.32 activation (Supplementary Table S3; Figure 5A). To determine which of the two proviruses may reflect the behavior of the *HERVH* subfamily as a whole, we looked for the effect of transcripts from all *HERVH* proviruses that were included in the selected 4844 EAC-specific transcripts. In addition to *HERVH Xp22*.32 and *HERVH 1p31.3*, another 6 *HERVH* proviruses were transcriptionally activated in EAC and this activation was more frequently associated with better survival, mirroring the effect of *HERVH Xp22*.32, although this association did not reach statistical significance in both OCCAMS EAC cohorts (Figure 5A). Moreover, expression of the different *HERVH* proviruses was not coordinated and for some was mutually exclusive (Figure 5B–F). Indeed, both in OCCAMS EAC and TCGA EAC samples, *HERVH Xp22*.32 was the most prominently expressed provirus, following by *HERVH 13q33.3*, whereas *HERVH 1p31.3* was only sporadically expressed (Figure 5B–D). The higher expression of *HERVH Xp22*.32, compared with other *HERVH* proviruses in EAC, was orthogonally confirmed using featureCounts (Supplementary Figure S10). Moreover, comparison of TCGA EAC and ESCC samples revealed a shift from *HERVH Xp22*.32 to *HERVH 13q33*.3 and *HERVH 1p31.3* expression (Figure 5C, D). Similarly to all EAC samples, TCGA COAD samples expressed most prominently *HERVH Xp22*.32 and only rarely *HERVH 1p31.3* (Figure 5E, F). This pattern of expression was also observed in gastrointestinal cancer cell lines from CCLE, where EAC cell lines are notably rare, with *HERVH Xp22*.32 expressed predominantly in adenocarcinomas, *HERVH 13q33*.3 expressed also in squamous cell carcinomas, and *HERVH 1p31.3* expressed more rarely (Figure 5G).

**Figure 6.**
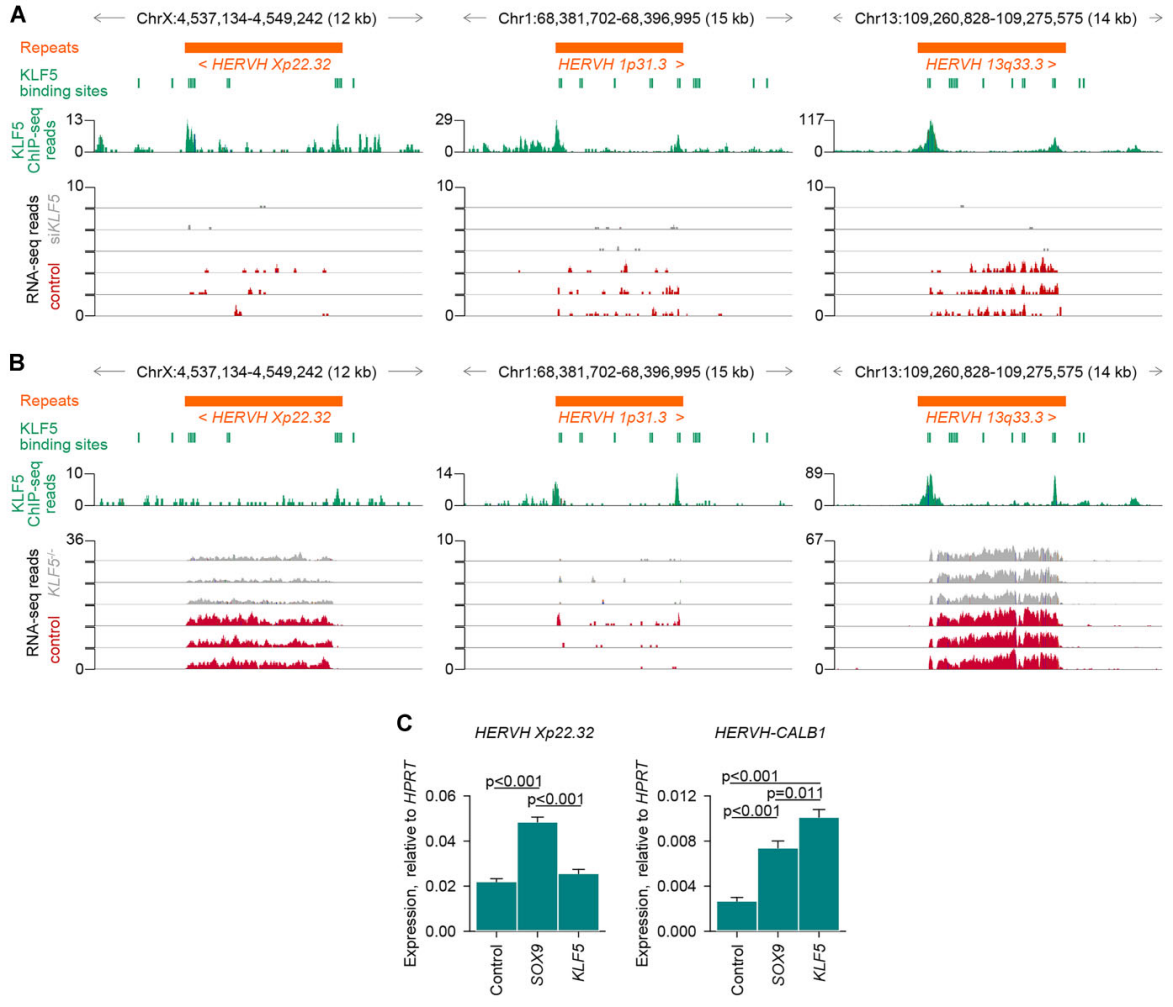
Regulation of individual *HERVH* proviruses by KLF5 and SOX9. (**A**) KLF5 ChIP-seq traces (green track) and RNA-seq traces of KLF5 knocked-down (si*KLF5*) and control EAC cells OE19 (three samples per group) (E-MTAB-8568; E-MTAB-8446) at the *HERVH Xp22*.32, *HERVH 1p31.3* and *HERVH 13q33.3* proviruses. (**B**) KLF5 ChIP-seq traces (green track) and RNA-seq traces of *KLF5*^−/−^ and control COAD cells HT-55 (three samples per group) (GSE147853; GSE147855) at the *HERVH Xp22*.32, *HERVH 1p31.3* and *HERVH 13q33.3* proviruses. Also indicated in (A) and (B) are KLF5 binding sites from the UCSC Genome Browser JASPAR Transcription Factor track. (**C**) Expression of *HERVH Xp22*.32 or *HERVH-CALB1*, relative to expression of *HPRT*, determined by RT-qPCR in EAC cells OE19 transfected to express SOX9 or KLF5, compared with control untransfected cells. Error bars represent the variation of two independent repeats each with three technical replicates and p values were calculated one way ANOVA with Bonferroni correction for multiple comparisons.

Disparate *HERVH* provirus expression within and between gastrointestinal cancers indicated independent regulation or responsiveness to transcription factors. Expression of stand-alone proviruses is driven by their LTRs. Through phyloregulatory analysis, *HERVH* LTR subfamilies have recently been reannotated (50), with *HERVH 13q33*.3 and *HERVH 1p31.3* belonging to the new LTR7u2 subfamily, whereas *HERVH Xp22*.32 belongs to the LTR7Y subfamily. Importantly, LTR7Y and LTR7u2 differ considerably in their responsiveness to transcription factors, particularly KLF5, which targets preferentially the LTR7Y subfamily (50,51). Consistent with earlier analyses (50,51), *HERVH Xp22*.32 LTR7Y LTRs contained twice as many consensus KLF5 binding sites than the LTR7u2 LTRs of the other two proviruses (Supplementary Figure S11). Differences between the proviruses, as well as between the 5′ and 3′ *HERVH Xp22*.32 LTRs, were also noted for SOX9 binding sites (Supplementary Figure S11). To validate the predicted effect of KLF5, we analyzed direct KLF5 binding to and expression of the three *HERVH* proviruses, using ChIP-Seq and RNA-seq data from the EAC and COAD cell lines OE19 and HT-55, respectively (52,53). Although the three proviruses were expressed at different levels in the two cell lines, KLF5 binding to the proviral LTRs was evident in all cases (Figure 6A, B). Moreover, loss of KLF5 activity reduced the expression of the three proviruses in both cell lines (Figure 6A, B)

Whilst these findings demonstrated that KLF5 was necessary for *HERVH* expression, they also indicated that it was not always sufficient. For example, despite high KLF5 activity in OE19 cells (52), *HERVH Xp22*.32 was modestly expressed (Figure 6A). Furthermore, overexpression of KLF5 in these cells did not raise *HERVH Xp22*.32 expression further (Figure 6C). As a control we examined another LTR7Y *HERVH* provirus in the *CALB1* locus, which we have recently found to be controlled by KLF5 in squamous lung cancer (51), and which readily responded to KLF5 overexpression in OE19 cells too (Figure 6C). Although KLF5 was not sufficient to induce *HERVH Xp22*.32 expression in OE19 cells, overexpression of SOX9 in these cells exhibited a significant effect (Figure 6C). Therefore, KLF5 or SOX9 exerted the strongest activating effect on all three proviruses examined. In contrast, loss of ARID1A, which was recently suggested to be responsible for overall *HERVH* activation in COAD (49), was rather specific to *HERVH 1p31.3*. Indeed, reanalysis of RNA-seq data from the COAD cell line HCT-116 (49), demonstrated that, in contrast to *HERVH 1p31.3*, which was strongly upregulated upon loss of ARID1A, *HERVH 13q33*.3 was downregulated and the remaining proviruses were either not expressed or not affected (Supplementary Figure S12). Collectively, these data highlight the independent regulation particularly of *HERVH Xp22*.32 and *HERVH 1p31.3*, which reconciles their contrasting association with EAC survival.

### *HERVH xp22.32* activation defines novel EAC molecular subtypes

As the dominant HERVH provirus expressed in EAC, we next explored whether the transcriptional activation of *HERVH Xp22.32* was associated with additional molecular features that could account for its association with better overall survival. Firstly, we examined if EAC subsets defined by high or low *HERVH Xp22.32* (using 1 TPM as the cut-off), matched EAC and ESCC subtypes described previously based on transcriptional profiles (54,55) or epigenetic changes (56). This analysis revealed only minimal overlap between *HERVH Xp22.32* and previously defined subsets (Supplementary Figure S13), suggesting that *HERVH Xp22.32* marked a distinct molecular process.

**Figure 7.**
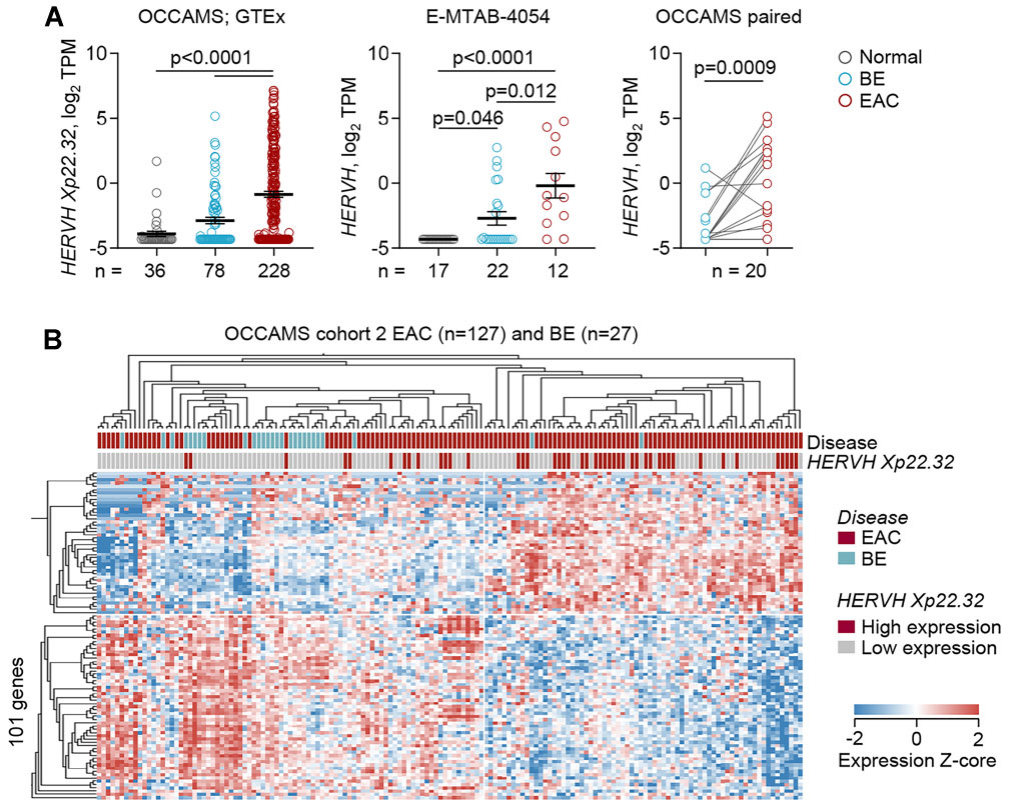
Expression of *HERVH Xp22*.32 in the progression to EAC. (**A**) Mean (±SEM) expression of *HERVH Xp22*.32 in GTEx normal esophagus and OCCAMS BE and EAC samples (left) and an independent dataset of normal esophagus and BE and EAC samples (E-MTAB-4054) (middle), and *HERVH Xp22*.32 expression in paired OCCAMs BE and EAC samples (right). Comparisons of the three types of tissue were carried out with Kruskal-Wallis tests with Dunn's correction for multiple comparisons, and of the paired samples with Wilcoxon matched-pairs signed rank test. (**B**) Heatmap of expression of 101 genes that were significantly (*q* < 0.05) differentially expressed between hierarchically clustered OCCAMS BE and EAC subsets according to *HERVH Xp22*.32 expression (using 1 TPM as the cut-off value to define high and low *HERVH Xp22.32* expression).

In the progressive stages leading up to EAC, *HERVH Xp22.32* was rarely or weakly activated in OCCAMS BE samples, but was more frequently and strongly activated in EAC (Figure 7A). Similar results were additionally obtained by analysis of an independent dataset of normal esophagus, BE and EAC samples (57) (Figure 7A). Moreover, in paired OCCAMS BE and EAC samples, *HERVH Xp22.32* was significantly upregulated in the latter (Figure 7A), suggesting that the progression of BE to EAC is characterized by *HERVH Xp22.32* activation in a substantial proportion of patients. However, EAC samples with high *HERVH Xp22.32* expression were transcriptionally more distant from BE samples than EAC samples with low *HERVH Xp22.32* expression were (Figure 7B), indicating that *HERVH Xp22.32* activation following BE progression to EAC is linked with a departure from the BE transcriptional profile.

**Figure 8.**
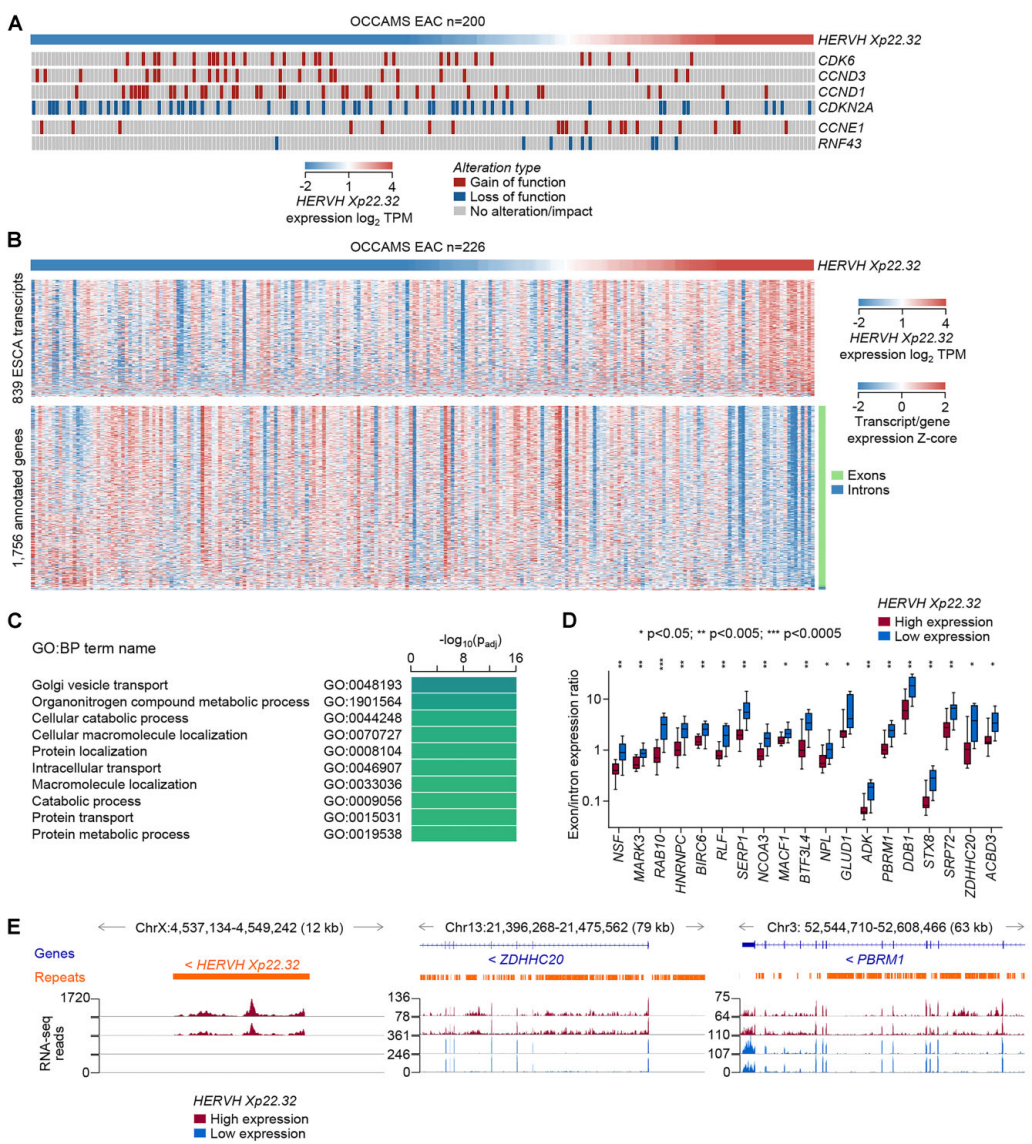
Molecular features of EAC subtypes defined by *HERVH Xp22.32* activation. (**A**) Driver gene alterations that correlated significantly (*P*< 0.05, *q* < 0.05) with *HERVH Xp22.32* expression by linear regression analyses. (**B**) Heatmap of expression of 839 ESCA-overexpressed assembled transcripts (top) and annotated exons and introns of 1756 annotated genes (bottom) that correlated significantly (*P*< 0.05, *q* < 0.05) with *HERVH Xp22.32* expression by linear regression analyses. (**C**) Functional annotation by gene ontology (GO) of the 1756 genes that correlated significantly with *HERVH Xp22.32* expression in (B). (**D**) Ratios of exon/intron expression in OCCAMS EAC samples with the highest and lowest *HERVH Xp22.32* expression (*n* = 10 per group) for 19 genes where the expression of exons and of intronic transcripts showed an inverse correlation with *HERVH Xp22.32* expression. *P* values were calculated using Student's *t*-tests. (**E**) RNA-seq traces of representative OCCAMS EAC samples with high or low *HERVH Xp22*.32 expression (two samples per group) at the *HERVH Xp22*.32, *ZDHHC20* and *PBRM1* loci.

Given that *HERVH Xp22.32* defined EAC subsets did not correspond to previously defined subsets, we investigate further characteristics. In the OCCAMS cohorts, *HERVH Xp22.32* high and low subsets had a similar total number of alterations in key driver genes (with 3.77 and 3.63 average number of altered driver genes per sample in each subset, respectively), as well as in a majority of these genes individually. However, significant differences (linear regression *P* < 0.05, *q* < 0.05) were observed for cell-cycle regulators, particularly in the balance of cyclin D and cyclin E alterations, previously associated with ESCC and EAC, respectively (9). Gain-of-function mutations in cyclin D subunits and loss-of-function mutations or deletions of its inhibitor *CDKN2A* were significantly enriched (linear regression *P* < 0.05, *q* < 0.05) in low *HERVH Xp22.32* samples (Figure 8A). In contrast, high *HERVH Xp22.32* samples had significantly more frequent gain-of-function mutations in cyclin E (Figure 8A).

Linear regression analyses identified 839 assembled transcripts, the expression of which was significantly (*P* < 0.05, *q* < 0.05) correlated with *HERVH Xp22.32* expression (Figure 8B). Importantly, a majority (833) of these transcripts, comprising predominantly aberrant or intronic contigs, were positive correlated with *HERVH Xp22.32* expression (Figure 8B), suggesting that the increased transcriptional diversity that characterized EAC is more pronounced in high *HERVH Xp22.32* samples. In contrast, transcriptional analysis of annotated genes (at the exon and intron level), identified 1756 genes, a vast majority of which, particularly the exons, were significantly downregulated in high *HERVH Xp22.32* samples (Figure 8B). Pathway analysis of the genes downregulated in samples with high *HERVH Xp22.32* expression indicated substantial alterations in metabolic and transport pathways (Figure 8C), implying cell-intrinsically reduced fitness associated with *HERVH Xp22.32* activation. The downregulated genes also included a smaller number of splicing and nucleosome factors (e.g. *CTCF*) (Supplementary Figure S14A), most of which were the same factors identified by the association with better EAC prognosis of their intronic RTE-overlapping transcripts (e.g. *CRNKL1* and *HNRNPU*) (Figure 4E). With the exception of IRF2, ISGs were notably absent from differentially expressed genes and deconvoluted immune cell signatures were also similar between *HERVH Xp22.32* high and low subsets, except for Treg cells and M2 macrophages, which were enriched in high and low *HERVH Xp22.32* samples, respectively (Supplementary Figure S14A, B).

Together, these findings suggested that transcriptional activation of *HERVH Xp22.32* was closely linked with incomplete RNA splicing, resulting in the EAC-characteristic increased representation of intronic RTEs, and parallel reduction in the expression of the fully-spliced, functional mRNA isoforms. Indeed, intersecting the genes with increased intronic but decreased exonic representation identified 19 genes where the exon/intron expression ratios were significantly lower in high than in low *HERVH Xp22.32* samples (Figure 8D). These were exemplified by *ZDHHC20*, an integral component of Golgi membrane involved in protein translation, and *PBRM1*, a chromatin remodeler, where mapping of RNA-seq reads demonstrated a shift in the coverage of introns at the expense of exons, in high *HERVH Xp22.32* samples (Figure 8E). In contrast, intronic reads were rare at the same loci in low *HERVH Xp22.32* samples (Figure 8E). These observations support a model whereby *HERVH Xp22.32* activation marks reduced expression of the functional isoforms of essential genes, owing to defective mRNA splicing.

## DISCUSSION

We have examined the transcriptional landscape of RTEs in EAC and described the origins and predictive value of its complexity. We found that incomplete RNA splicing affects both EAC and ESCC, and is shared with STAD, OV and COAD. In an independent analysis of the number of cancer-specific exon-exon junctions, OV stood out among all cancer types followed by liver hepatocellular carcinoma (LIHC), ESCA and STAD (58). Similarly, OV, ESCA and STAD were the top 3 cancer types in an analysis of cryptic introns (59), although many of them may have been cDNA synthesis artifacts (44). Collectively, these studies underscore the aberrant splicing patterns observed repeatedly in ESCA and related STAD.

Incomplete removal of introns will elevate the representation of RTEs, which are found in abundance in intronic regions, and give the impression of an increase in independent RTE transcription (60). Libraries prepared from rRNA-depleted RNA, such as those from the OCCMAS cohorts, may be enriched for incompletely processed or unprocessed pre-mRNAs, with the potential to distort the representation of intronic repeats (61). However, it is unlikely that incomplete RNA splicing reported here for EAC resulted from the use of rRNA-depleted RNA libraries for the following reasons. Firstly, the original *de novo* transcriptome assembly employed only poly(A)-selected RNA data from TCGA cohorts (8) and therefore assembled contigs would have to be present in poly(A)-selected RNA. Secondly, the expression comparisons between distinct cancer types and respective healthy tissues that identified the selected 4844 ESCA-overexpressed transcripts were also carried out using only poly(A)-selected RNA data from TCGA and GTEx, and they would not be affected by increased abundance of intronic reads in rRNA-depleted RNA. Lastly, an increase in intronic read abundance in ESCA, compared with most other cancer types and healthy tissues, was revealed using only poly(A)-selected RNA data with identical methodology.

The presence of AT-rich sequences naturally flanking non-LTR retroelement integrations and the use of poly(A) priming during cDNA library preparation may also generate cDNA from seemingly polyadenylated retroelement RNA. This may also give the impression of independent RTE transcription in cases of incomplete intron removal such as in EAC and other cancers, as well as in intron retention seen in physiological conditions (43). For example, the recent association of a MER4 retroelement on Chr6 p22.3 with the outcome of check-point blockade in non-small cell lung cancer is likely due to incomplete removal of the first intron of *ACOT13* locus where this retroelement resides (62). Similarly, an orthogonal k-mer approach for analysis of RTE transcription in lung adenocarcinoma identified numerous differentially expressed intronic contigs likely resulting from differential transcription and incomplete splicing of the encompassing genes (63). Nevertheless, certain intronic RTEs may play a more active role in the aberrant splicing of the introns that contain them, than being mere passengers. Indeed, the presence of recently integrated non-LTR retroelements, particularly *Alu* elements, has been reported to influence splicing of the intron in which they reside in multiple tissues (64–66).

Failure to remove introns and intronic RTEs may have direct and indirect consequences for cellular fitness. Although accumulation of RTE transcripts has been linked with induction of cell-intrinsic antiviral responses, characterized by IFN production, in multiple other cancers (5), we found no obvious IFN signature associated with aberrant splicing in EAC. Alternative splicing has been reported to affect ESCC and EAC differentially, also depending on the individual gene, with more alternative splicing events correlating with better than with worse prognosis (47,67). Proteomics analyses indicated specific upregulation of spliceosome components, including *CRNKL1* and *HNRNPU*, in the transition from BE to EAC (68), as well as in ESCC (69), likely reflecting inadequate compensatory increase. Moreover, splicing and nucleosome factors, including *CRNKL1*, *HNRNPU* and *HNRNPL* were also found here to affect EAC survival, in agreement with previous reports (47,67). As these common processes of incomplete RNA splicing were responsible for the generation of a majority of the ESCA-overexpressed transcripts identified here, it is perhaps expected that they individually correlate with better prognosis. This phenotype, which is most pronounced in EAC, strongly links with transcriptional activation of *HERVH Xp22.32*. This provirus is one of several stand-alone RTEs that are transcriptionally activated in a highly cancer-specific manner. Of note, *HERVH Xp22.32* activation is mutually exclusive with activation of the *L1PA2-L1PB1* elements at the *GNGT1* locus, which is also cancer-specific, indicating the existence of EAC subsets. Similarly to aberrant splicing, we found that *HERVH Xp22.32* activation predicts better EAC prognosis.

While activation of the *HERVH Xp22.32* provirus has long been recognized as a hallmark of COAD (45,48), its potential significance had not been fully investigated. *HERVH* expression in COAD was implicated in chemokine production and recruitment of mesenchymal stem cells and myeloid-derived suppressor cells, thereby exerting a protumoral effect (70). However, the particular provirus that was studied was a *HERVH* integration on Chr 3q26, chosen for the presence of a relatively intact *env* open reading frame (70). More recently, activation of *HERVH* more broadly, attributed to loss of the repressor *ARID1A*, was suggested to promote COAD progression (49). However, the effect of *ARID1A* loss appears restricted primarily to *HERVH 1p31.3*, a provirus we also associate with worse EAC prognosis, in agreement with findings in COAD (49), but also a provirus that is rarely expressed in EAC or indeed in COAD. Further confounding the involvement of individual *HERVH* proviruses, the strategies employed for knockdown of *HERVH* expression in these studies (49,70), target multiple proviruses in other chromosomal locations but only variably *HERVH Xp22.32*.

Activation of *HERVH Xp22.32* and associated aberrant RNA processing in EAC does not correlate well with previously defined EAC subsets (54–56), but instead marks subtypes with distinct molecular features, such as enrichment for cyclin E, rather than cyclin D alterations. This finding suggests commonalities between EAC samples with low *HERVH Xp22.32* expression and ESCC samples, which are also enriched in cyclin E alterations (9). This notion is further supported by mutually exclusive expression of *HERVH Xp22.32* and of the novel *L1PA2-L1PB1* transcript at the *GNGT1* locus, which is also a characteristic of ESCC. Furthermore, compared with EAC, ESCC expresses higher levels of *HERVH 13q33.3* than of *HERVH Xp22.32* and this balance also distinguishes EAC subsets. Together, these observations indicate that EAC samples with high *HERVH Xp22.32* expression retain more pronounced adenocarcinoma characteristics, are less similar to the their BE precursor, exhibit defective RNA splicing, and predict better prognosis.

Elucidating the precise reasons for the association of *HERVH Xp22.32* activation with these phenotypes are not understood at present. It is possible that high *HERVH Xp22.32* expression is not simply a marker for the underlying processes, but it is actively involved through provision of RNA scaffolds for transcription factors, as suggested by studies in human embryonic stem cells (71,72), or production of biological active protein products. While these non-exclusive mechanisms remain to be elucidated, the present study establishes the association of *HERVH Xp22.32* in particular, and of the unique RTE transcriptional landscape of EAC more generally, with its subtypes and prognosis.

## Supplementary Material

zcad040_Supplemental_Files

## Data Availability

The RNA-seq and WGS data used during this study have been deposited at the European Genome-Phenome Archive (EGA), which is hosted by The European Bioinformatics Institute (EBI) and the Centre for Genomic Regulation (CRG), under the accession numbers EGAD00001011076 (RNAseq) and EGAD00001011095 (WGS). Access is controlled by the Data Access Committee. Details on how to apply for access are available at https://docs.icgc-argo.org/docs/data-access/daco/applying. TCGA and GTEx data used for the analyses described in this manuscript were obtained from dbGaP (https://dbgap.ncbi.nlm.nih.gov) accession numbers phs000178.v10.p8.c1 and phs000424.v7.p2.c1 in 2017. Other publicly available dataset supporting the findings of this study included the following: RNA-seq samples from normal esophagus, BE and EAC (E-MTAB-4054) (57). ISO-seq data from ESCC cell lines KYSE140, KYSE510, SHEEC and TE5 and normal immortalized esophageal squamous epithelial cell line SHEE were (PRJNA515570) (42). Direct RNA-seq (SRR14326971) and direct cDNA-seq (SRR14326972) from HEK293T cells (44). KLF5 ChIP-seq (E-MTAB-8568) and RNA-seq from control and *KLF5* knocked-down EAC cells OE19 (E-MTAB-8446) (52). KLF5 ChIP-seq (GSE147853) and RNA-seq from control and *KLF5* knocked-out COAD cells HT-55 (GSE147855) (53). RNA-seq from control and *ARID1A* knocked-out COAD cells HCT-116, with or without *HERVH* knock-down (GSE180475) (49).

## Notes

Present address: Jan Attig, Pharma Research and Early Development, F. Hoffmann-La Roche, Basel, Switzerland.

Present address: George R. Young, Bioinformatics and Computing Facility, MRC London Institute of Medical Sciences, London, UK.

## APPENDIX

**Oesophageal Cancer Clinical and Molecular Stratification (OCCAMS) Consortium**

Rebecca C. Fitzgerald^6^, Paul A. W. Edwards^5,6,8^, Nicola Grehan^6^, Barbara Nutzinger^11^, Elwira Fidziukiewicz^11^, Aisling M. Redmond^11^, Sujath Abbas^11^, Adam Freeman^11^, Elizabeth C. Smyth^12^, Maria O’Donovan^11,13^, Ahmad Miremadi^11,13^, Shalini Malhotra^11,13^, Monika Tripathi^11,13^, Calvin Cheah^11^, Hannah Coles^11^, Connor Flint^11^, Matthew Eldridge^5^, Maria Secrier^5^, Ginny Devonshire^5^, Sriganesh Jammula^5^, Jim Davies^14^, Charles Crichton^14^, Nick Carroll^12^, Richard H. Hardwick^12^, Peter Safranek^12^, Andrew Hindmarsh^12^, Vijayendran Sujendran^12^, Stephen J. Hayes^15,16^, Yeng Ang^15,17,18^, Andrew Sharrocks^18^, Shaun R. Preston^19^, Izhar Bagwan^19^, Vicki Save^20^, Richard J. E. Skipworth^20^, Ted R. Hupp^21^, J. Robert O’Neill^12,20,21^, Olga Tucker^10,22^, Andrew Beggs^9,10^, Philippe Taniere^10^, Sonia Puig^10^, Gianmarco Contino^9,10,38^, Timothy J. Underwood^23,24^, Robert C. Walker^23,24^, Ben L. Grace^23^, Jesper Lagergren^25,26^, James Gossage^22,25^, Andrew Davies^22,25^, Fuju Chang^22,25^, Ula Mahadeva^25^, Vicky Goh^22^, Francesca D. Ciccarelli^3,4^, Grant Sanders^27^, Richard Berrisford^27^, David Chan^27^, Ed Cheong^28^, Bhaskar Kumar^28^, L. Sreedharan^28^, Simon L. Parsons^29^, Irshad Soomro^29^, Philip Kaye^29^, John Saunders^15,29^, Laurence Lovat^30^, Rehan Haidry^30^, Michael Scott^31^, Sharmila Sothi^32^, Suzy Lishman^2^, George B. Hanna^33^, Christopher J. Peters^33^, Krishna Moorthy^33^, Anna Grabowska^34^, Richard Turkington^35^, Damian McManus^35^, Helen Coleman^35^, Russell D. Petty^36^ & Freddie Bartlett^37^

^8^Department of Pathology, University of Cambridge, Cambridge, UK.

^9^Institute of Cancer and Genomic Sciences, College of Medical & Dental Sciences, University of Birmingham, Birmingham, UK.

^10^University Hospitals Birmingham NHS Foundation Trust, Birmingham B15 2GW, UK.

^11^Department of Surgery, University of Cambridge, Cambridge, UK.

^12^Cambridge University Hospitals NHS Foundation Trust, Cambridge CB2 0QQ, UK.

^13^Department of Histopathology, Addenbrooke's Hospital,Cambridge, UK.

^14^Department of Computer Science, University of Oxford, Oxford OX1 3QD, UK.

^15^Salford Royal NHS Foundation Trust, Salford M6 8HD, UK.

^16^Faculty of Medical and Human Sciences, University of Manchester, Manchester M13 9PL, UK.

^17^Wigan and Leigh NHS Foundation Trust, Wigan, Manchester WN1 2NN, UK.

^18^GI Science Centre, University of Manchester, Manchester M13 9PL, UK.

^19^Royal Surrey County Hospital NHS Foundation Trust, Guildford GU2 7XX, UK.

^20^Edinburgh Royal Infirmary, Edinburgh EH16 4SA, UK.

^21^Edinburgh University, Edinburgh EH8 9YL, UK.

^22^King's College London, London WC2R 2LS, UK.

^23^University Hospital Southampton NHS Foundation Trust, Southampton SO16 6YD, UK.

^24^Cancer Sciences Division, University of Southampton, Southampton SO17 1BJ, UK.

^25^Guy's and St Thomas's NHS Foundation Trust, London SE1 7EH, UK.

^26^Karolinska Institute, Stockholm SE-171 77, Sweden.

^27^Plymouth Hospitals NHS Trust, Plymouth PL6 8DH, UK.

^28^Norfolk and Norwich University Hospital NHS Foundation Trust, Norwich NR4 7UY, UK.

^29^Nottingham University Hospitals NHS Trust, Nottingham NG7 2UH, UK.

^30^University College London, London WC1E 6BT, UK.

^31^Wythenshawe Hospital, Manchester M23 9LT, UK.

^32^University Hospitals Coventry and Warwickshire NHS Trust, Coventry CV2 2DX, UK.

^33^Department of Surgery and Cancer, Imperial College, London W2 1NY, UK.

^34^Queen's Medical Centre, University of Nottingham, Nottingham, UK.

^35^Centre for Cancer Research and Cell Biology, Queen's University Belfast, Belfast BT7 1NN, Northern Ireland.

^36^Tayside Cancer Centre, Ninewells Hospital and Medical School, Dundee DD1 9SY, Scotland.

^37^Portsmouth Hospitals NHS Trust, Portsmouth PO6 3LY, England.
