# Supplementary material for: The transcriptional landscape of endogenous retroelements delineates esophageal adenocarcinoma subtypes
Source: NAR Cancer. 2023 Jul 26;5(3):zcad040. doi: 10.1093/narcan/zcad040 (PMC10370457; doi:10.1093/narcan/zcad040)
Supplement: zcad040_Supplemental_Files [file zcad040_supplemental_files.zip › Supplementary figures_r1_clean.pdf]

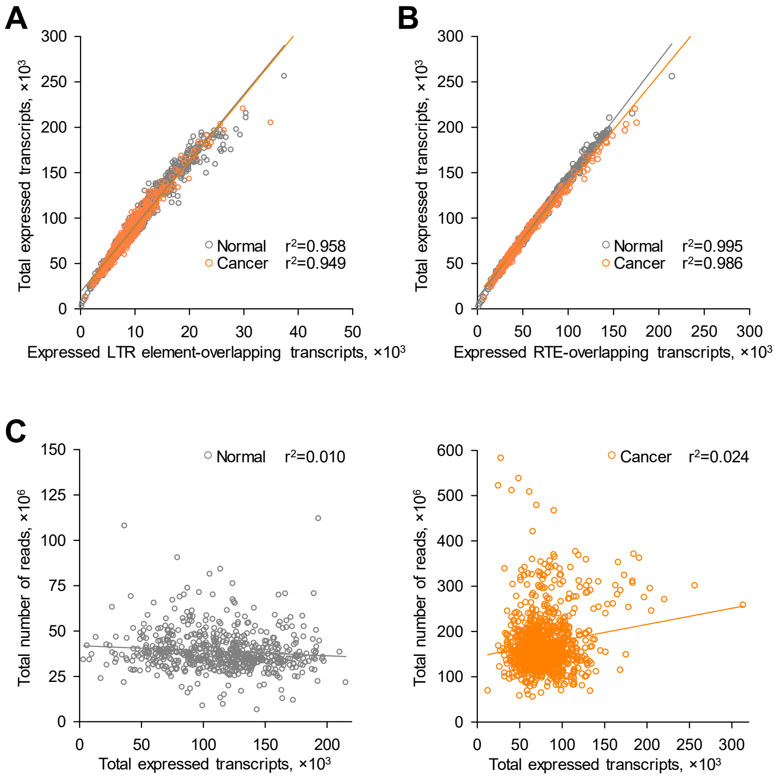

Figure S1. Correlation between expressed transcripts according to overlap with RTEs. (**A**) Correlation between LTR element-overlapping transcripts and total number of transcripts expressed (≥0.5 TPM) in cancer (n=768) or normal tissue samples (n=811). (**B**) Correlation between RTE-overlapping transcripts and total number of transcripts expressed in the same samples. (**C**) Correlation between library size (plotted as the total number of sequenced reads) and total number of transcripts expressed in normal samples from GTEx (left) and cancer samples from TCGA (right). Note that TCGA samples are sequenced at greater depth than GTEx samples. Symbols represent individual samples and r values denote the Spearman correlation coefficients.

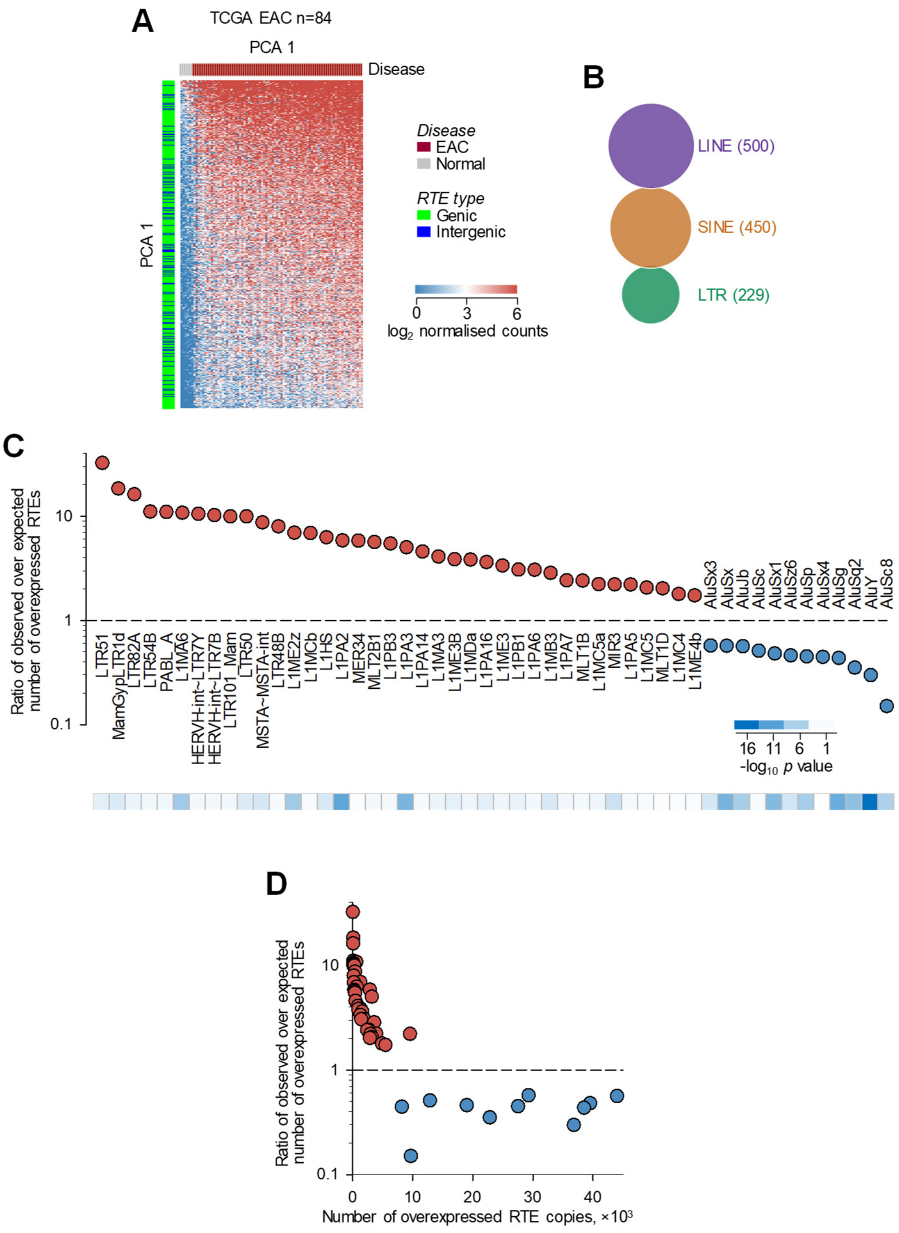

Figure S2. Increased inclusion of RTEs in the EAC transcriptome. (**A**) Heatmap of expression of 1,179 RTEs that were found significantly overexpressed (>6-fold change, p<0.05, q<0.05) in EAC (n=78) compared with normal esophagus samples (n=6) from TCGA by featureCounts. Both samples and RTEs are plotted according to Principal Component Analysis (PCA) component 1. (**B**) Proportion of LINEs, SINEs and LTR elements in the 1,179 EAC-overexpressed RTEs. (**C**) Enrichment of the indicated RTE subfamily in the 1,179 EAC-overexpressed RTEs, compared with all expressed RTEs (p values were calculated with Fisher's exact tests). (**D**) Correlation between enrichment of RTE subfamilies and the copies of subfamily members in the transcriptome.

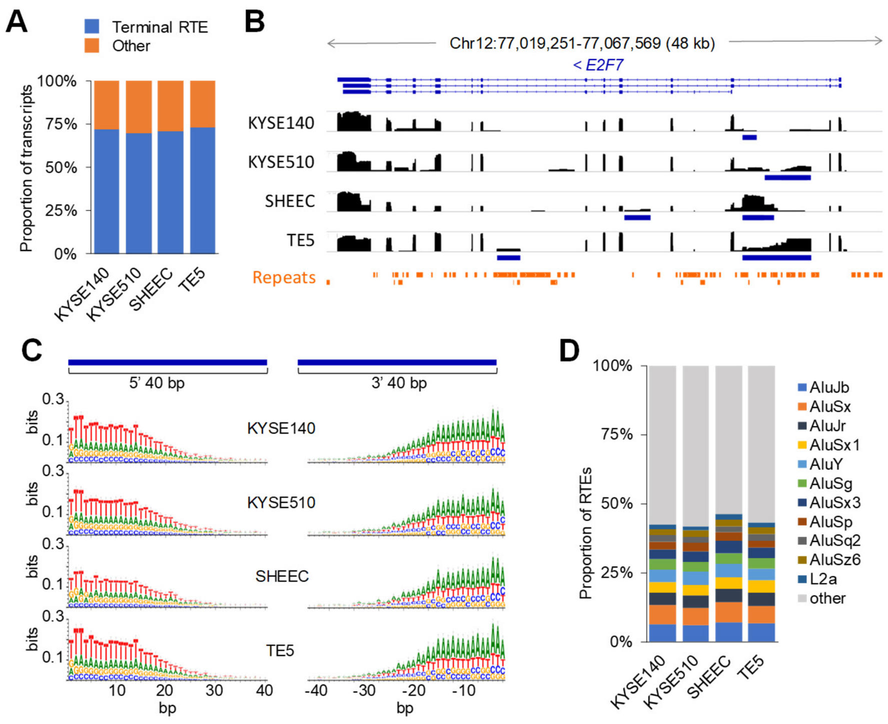

Figure S3. Properties of intronic contigs specific to the EAC transcriptome. (**A**) Proportion of transcripts with a terminal RTE or an internal RTE (other) identified in ISO-seq data from the ESCC cell lines KYSE140, KYSE510, SHEEC and TE5 (PRJNA515570). (**B**) Example of ISO-seq read mapping from the same cell lines to the *E2F7* locus. Blue bars indicate fully intronic contigs. RTEs are also indicated in the orange track (Repeats). (**C**) Logo plots for the 40 bp at the 5' and 3' ends of all intronic reads from the same cell lines. (**D**) Proportion of the indicated RTE families at the 5' or 3' ends of all intronic reads from the same cell lines.

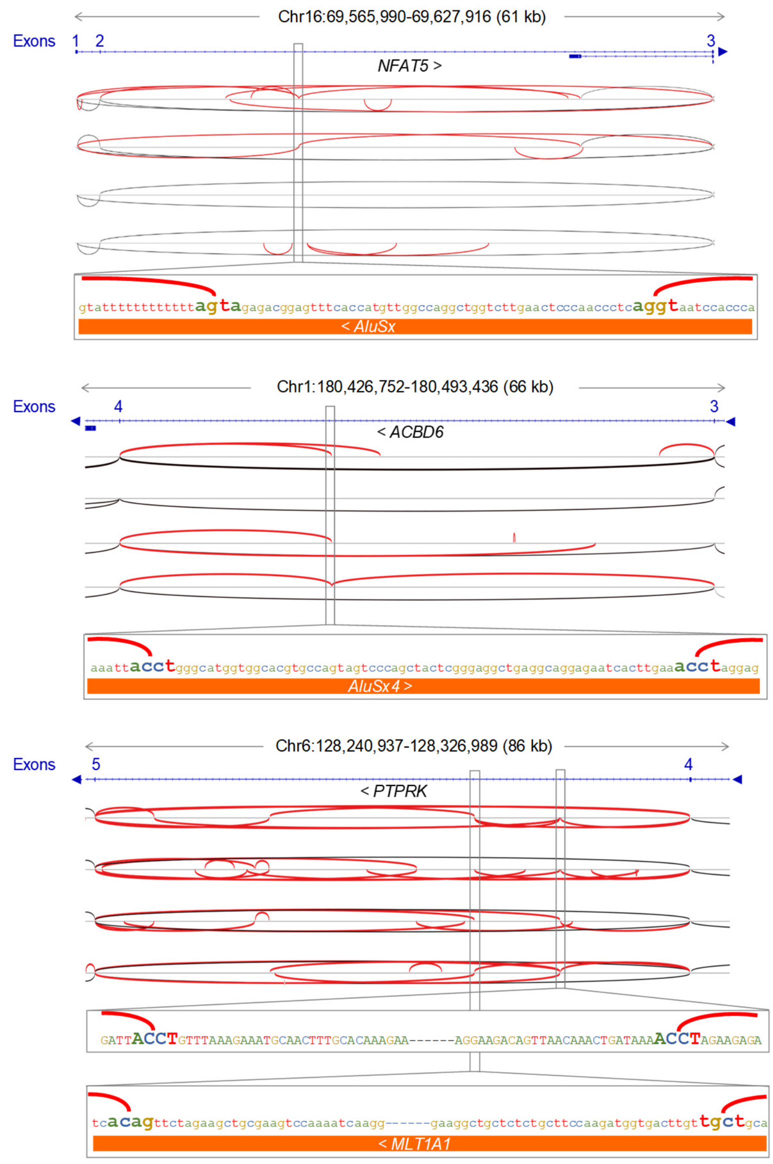

Figure S4. Splicing between intronic RTEs and flanking exons in EAC. Examples of splicing between annotated exons and intronic RTEs in the *NFAT5*, *ACBD6* and *PTPRK* genes in 4 representative OCCAMS EAC samples. Only part of each gene is shown. Black and red lines in sashimi plots indicate annotated and unannotated splicing evens, respectively. Insets show the nucleotide sequence at the splicing locations (denoted in larger font size) found in close proximity within each repeat.

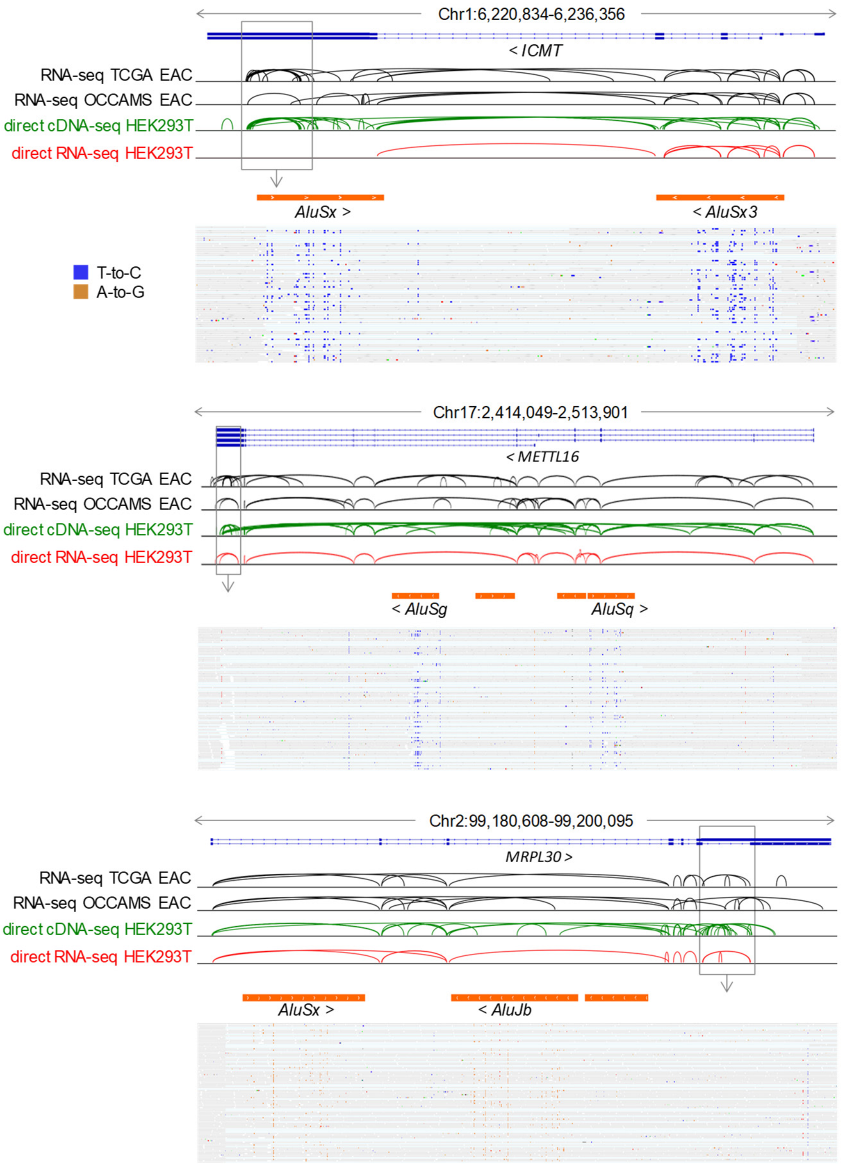

Figure S5. Splicing events in 3' UTRs harboring inverted *Alu* repeats in EAC. Examples of artifactual (*ICMT*) and actual splicing (*METTL16* and *MRPL30*) in the 3' UTR of the indicated genes. Sashimi plots include TCGA and OCCAMS EAC RNA-seq samples (black), and direct cDNA-seq (green) (SRR14326972) and direct RNA-seq (red) (SRR14326971) from HEK293T cells. Insets depict the inverted *Alu* repeats and mapped reads from OCCAMS EAC samples highlighting ADAR-mediated A-to-I editing (as I is recognized by the cellular machinery as G, A-to-I editing is converted to A-to-G and T-to-C on the positive and negative strand, respectively).

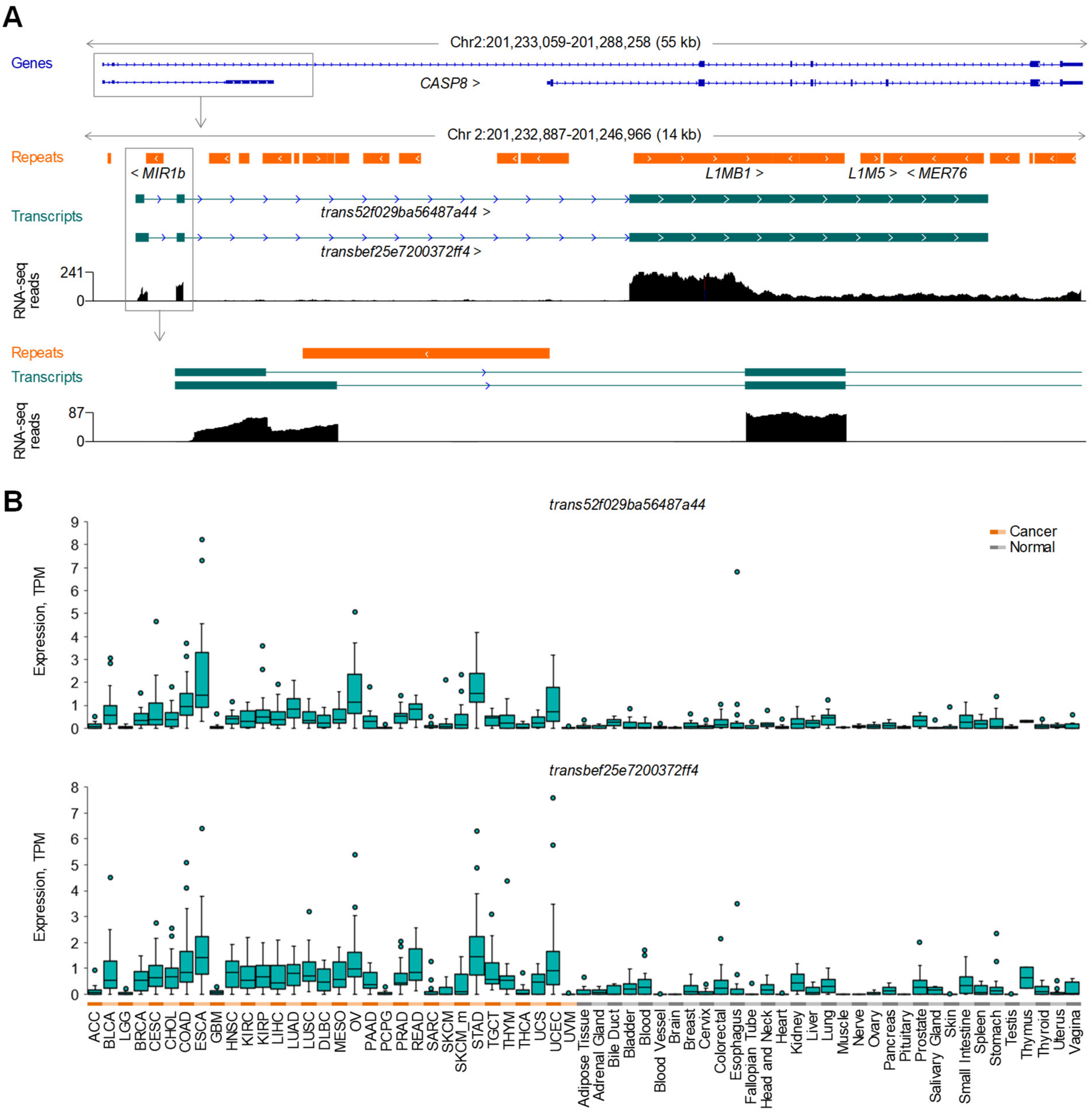

Figure S6. Cancer-specific, RTE-overlapping transcripts at the *CASP8* locus. (**A**) GENCODE annotated transcripts (Genes), RTEs (Repeats), assembled cancer-specific, RTE-overlapping transcripts (CETs), and RNA-seq traces of 24 combined TCGA ESCA samples. This locus transcribes an additional two isoforms that use several intronic RTEs as an alternative third and terminal exon. One of these (matching the Ensembl annotated transcript ENST00000490682.5) comprises a longer first exon through alternative splicing into an intronic *MIR1b* element. The inset shows the two distinct transcripts in relation to the exonised *MIR1b* element. (**B**) Expression in TPM of the two transcripts in TCGA (n=24 per cancer type) and GTEx samples (n=2-156 per tissue type). Box plots denote median value and quartiles, whiskers denote 1.5x the interquartile range, and individual points denote outliers.

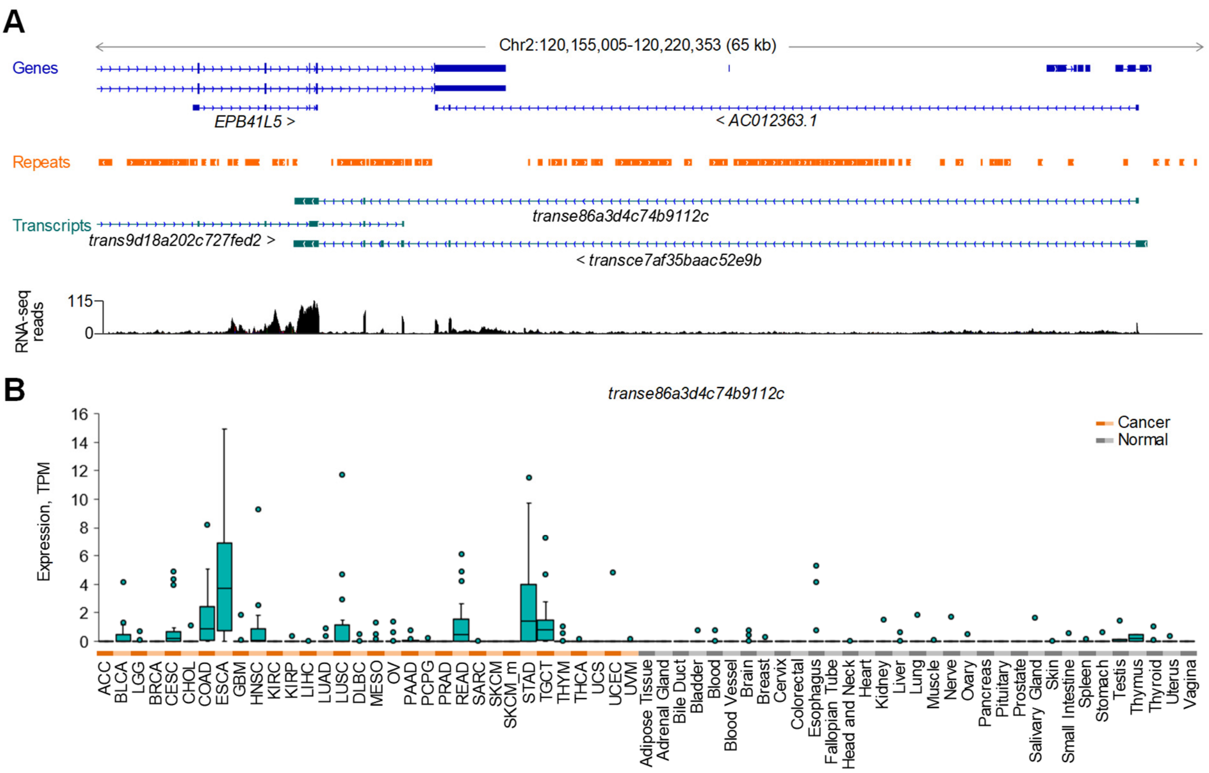

Figure S7. Cancer-specific, RTE-overlapping transcripts at the *EPB41L5* locus. (**A**) GENCODE annotated transcripts (Genes), RTEs (Repeats), assembled cancer-specific, RTE-overlapping transcripts (CETs), and RNA-seq traces of 24 combined TCGA ESCA samples. Several novel exons are created by exonisation of RTEs in the last intron of the *EPB41L5* gene. (**B**) Expression in TPM of the indicated transcript in TCGA (n=24 per cancer type) and GTEx samples (n=2-156 per tissue type). Box plots denote median value and quartiles, whiskers denote 1.5x the interquartile range, and individual points denote outliers.

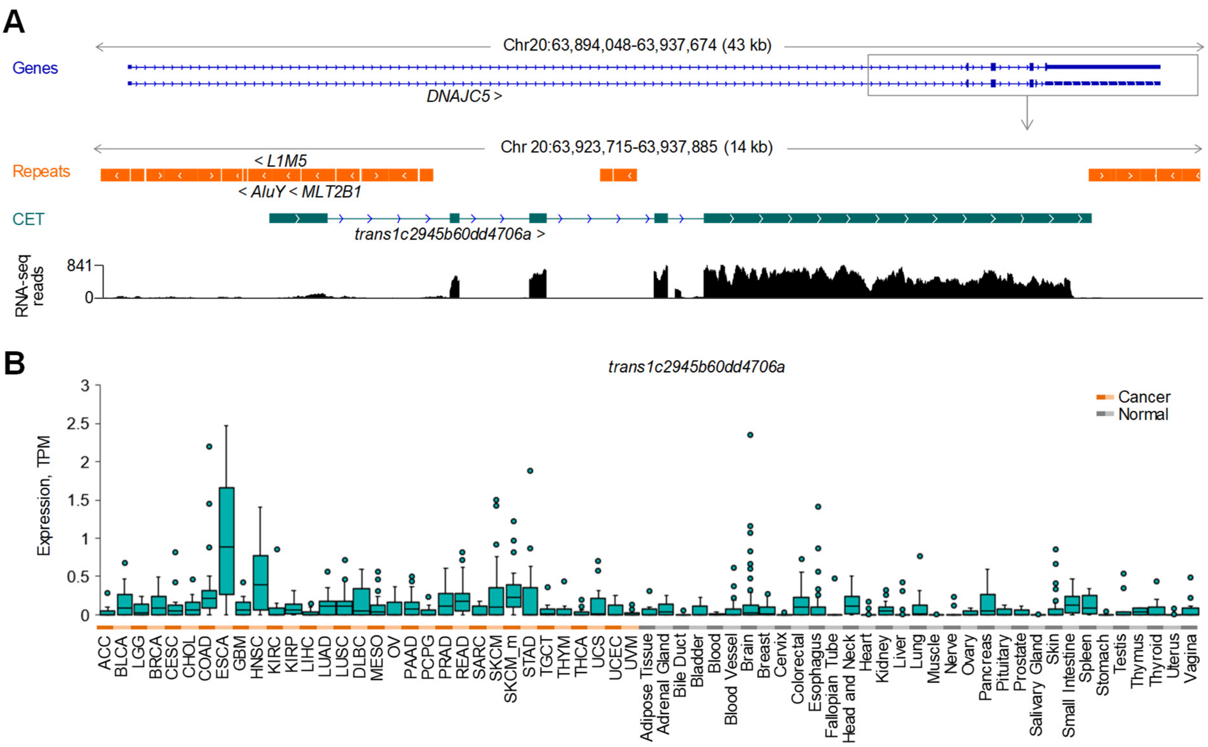

Figure S8. Cancer-specific, RTE-overlapping transcripts at the *DNAJC5* locus. (**A**) GENCODE annotated transcripts (Genes), RTEs (Repeats), assembled cancer-specific, RTE-overlapping transcripts (CETs), and RNA-seq traces of 24 combined TCGA ESCA samples. Alternative promoter activity of RTEs in the first intron of the *DNAJC5* gene (inset) creates an ESCA-upregulated isoform, retaining protein-coding potential and matching the RefSeq predicted transcript XM_011529048.2. (**B**) Expression in TPM of the indicated transcript in TCGA (n=24 per cancer type) and GTEx samples (n=2-156 per tissue type). Box plots denote median value and quartiles, whiskers denote 1.5x the interquartile range, and individual points denote outliers.

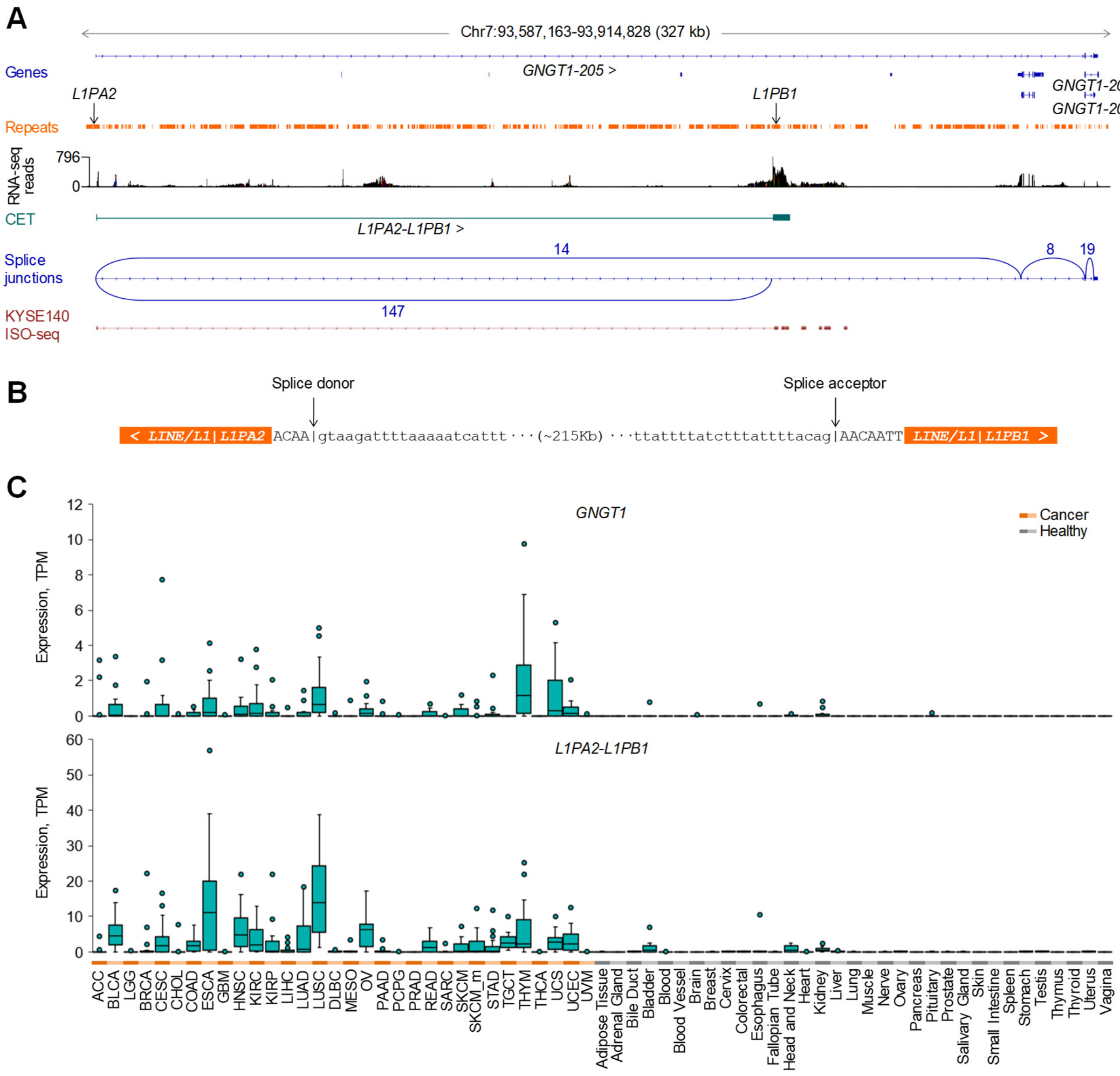

Figure S9. Cancer-specific, RTE-overlapping transcripts at the *GNGT1* locus. (**A**) GENCODE annotated transcripts (Genes), RTEs (Repeats), RNA-seq traces of 24 combined TCGA ESCA samples, assembled cancer-specific RTE-overlapping transcripts (CETs), splice junctions of a representative TCGA ESCA sample (minimum cut-off of 8 junctions), and contigs in ISO-seq data from the ESCC cell line KYSE140 (PRJNA515570). (**B**) Splice junction between the upstream *L1PA2* element (on the negative strand) and the downstream *L1PB1* element (on the positive strand), verified by RT-PCR amplification and Sanger sequencing. (C) Expression in TPM of the annotated *GNGT1* and the novel *L1PA2-L1PB1* transcripts in TCGA (n=24 per cancer type) and GTEx samples (n=2-156 per tissue type). Box plots denote median value and quartiles, whiskers denote 1.5x the interquartile range, and individual points denote outliers.

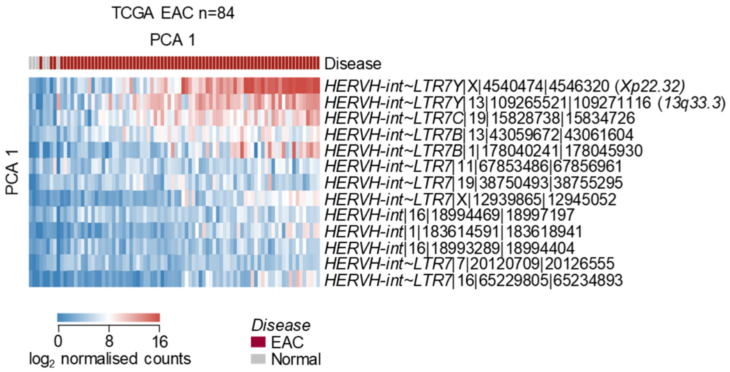

Figure S10. Pattern of *HERVH* expression in EAC quantified using featureCounts. Heatmap of expression of the indicated *HERVH* proviruses in EAC (n=78) and normal esophagus samples (n=6) from TCGA, quantified using featureCounts. Both samples and *HERVH* proviruses are plotted according to PCA component 1. The annotation of HERVH proviruses is as follows: subfamily|chr|start position|end position in GRCh38/hg38. *HERVH Xp22.32* and *13q33.3* are also indicated in parentheses.

A

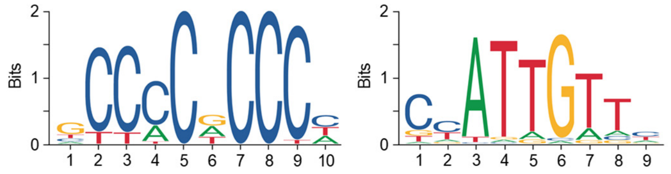

B

KLF5 preferred bases in all positions
KLF5 second preference in one position
SOX9 second preference in two positions

>*HERVH Xp22.32* LTR7Y.5′
TGTCAGGCCTCTGAGCCCAAGCCAAGCCATCGCATCCCCTGTGACTTGCATGTATACGCCCAGATGGCCTGAAGTAACTGAAG
AATCACAAAAGAAGTGAATATGCCCTGCCCCACCTTAACTGATGACATTCCACCATTGTGATTTGTTCCTGCCCCACCCTAAC
TGATCAATGTACTTTGTAATCTCCCCCACCCTTAAGAAGGTCCTTTGTAATTCTCCCCACCCTTGAGAATGTACTTTGTGAGA
TCCACCCCTGCCCACCAGAGAACAACCCCCTTTGACTGTAATTTTCCATTACCTTCCCAAATCCTATAAAACGGCCCCACCCC
TATCTCCCTTTGCTGACTCTCTTTTCGGACTCAGCCCGCCTGCACCCAGGTGAAATAAACAGCCATGTTGCTCACACAAAGCC
TGTTTGGTGGTCTCTTCACATGGACGCACATGAAA

>*HERVH Xp22.32* LTR7Y.3′
TGTCAGGCCTCTGAGCCCAGGCCAGGCCATCGCATCCCCTGTGACTTGCACGTATACATCCAGATGGCCTAAAGTAACTGAAG
ATCCACAAAAGAAGTAAAAACAGCCTTAACTGATGACATTCCAACATTGTGATTTGTTCCTGCCCCACCCTAACTGATAAATG
TACTTTGTAATCTCCCCCACCCTTAAGAAGGTCCTTTGTAATTCTCCCCACCCTTGAGAGTGTACTTTGTGAGATCCACACCT
GCCCACCAGAGAACAAACCCCCTTTGACTGTAATTTTCCATTACCTTCCCTAATCCTATAAAACGGCCCCACCCCATCTCCCT
TTGCTGACTCTCTTTTCGGACTCAGCCCGCCTGCACCCAGGTGAAATAAACAGCCTTGTTGCTCACACAAAGCCTGTTTGGTG
GTCTCTTCACACGGACGCGCGTGAAA

>*HERVH 1p31.3* LTR7u2.5′
TGTCAGGCCTCTGAGCCCAAGTCAAACCATCGCATCCCCTGTGACTTGCATGTATACGCCCAGATGGCCTGAAGTAACTGAAG
AATCACAAAAGAAGTGAAAAGGCCCTGCCCCGCCTTAACTGATGACATTCCACCATTGTGATTTGTTCCTGCCCCACCTTAAC
TGAGTGATTAACCCTGTGAATTTCCTTCTCCTGGCTCAGAAGCTCCCCCACTGAGCACCTTGTGACCCCCGCCCCTGCCCACC
AGAGAACAACCCCCTTTGACTGTAATTTTCCATTACCTTCCCAAATCCTATATAAGACGGCCCCACCCTTATCTCCCTTCGCT
GACTCTCTTTTCGGACTCAGCCCACCTGCACCCAGGTGAAATAAACAGCTTTATTGCTCACACAAAGCCTGTTTGGTGGTCT

>*HERVH 1p31.3* LTR7u2.3′
TGTCAGGCCTCTGAGCCCAAGCCAAGCCATCGCATCCCCTGTGACTTGCATGTATACGCTCAGATGGCCTGAAGTAACTGAAG
AATCACAAAAGAAGTGAAAAGGCCCTGCCCCGCCTTAACTGATGACATTCCACCATTGTGATTTGTTCCTGCCCCACCTTAAC
TGAGTGATTAACCCTGTGAATTTCCTTCTCCTGGCTCAGAAGCTCCCCCACTGAGCACCTTGTGACCCCCGCCCCTGCCCACC
AGAGAACAACCCCCTTTGACTAATTTTCCATTACCTTCCCAAATCCTATAAGATGGCCCCACCCTTATCTCCCTTCGCTGACT
CTCTTTTCGGACTCAGCCCACCTGCACCCAGGTGAAATAAATAGCTTTATTGCTCACACAAAGCCTGTTTGGTGGTCTCTTCA
CACGGACGCGCATGAAA

>*HERVH 13q33.3* LTR7u2.5′
TGTCAGGCCTCTGAGCCCAAGCCAAGCCATCGCATCCCCTGTGACTTGCACGTATACGCCCAGATGGCCTGAAGTAACTGAAG
AATCACAAAAGAACTGAAAAGGCCCTGCCCCGCCTTAACTGATGACATTCCACCATGGTGATTTGTTCTTGCCCCACCTTAAC
TGAGTGATTAACCCTGTGAATTTCCTTCTCCTGGCTCAGCAGCTCCCCCACTGAGCACCTTGTGACCCCCGCCCCTGCCCACC
AGAGAACAACCCCCTTTGACTGTAATTTTCCATTACCTTCCCAAATCCTATAAAACGGCCCCACCCCATCTCCCTTCGCTGAC
TCTCTTTTCGGACTCAGCCCGCCTGCCCCCAGGTGAAATAAACAGCCATGTTGCTCACACAAAGCCTGTTTGGTGTCTC

>*HERVH 13q33.3* LTR7u2.3′
TGTCAGGCCTCTGAGCCCAAGCCAAGCCATCGCATCCCCTGTGACTTGCACGTATACGCCCAGATGGCCTGAAGTAACTGAAG
AATCACAAAAGAACTGAAAAGGCCCTGCCCCGCCTTAACTGATGACATTCCACCATGGTGATTTGTTCTTGCCCCACCTTAAC
TGAGTGATTAACCCTGTGAATTTCCTTCTCCTGGCTCAGAAGCTCCCCCACTGAGCACCTTGTGACCCCCGCCCCTGCCCACC
AGAGAACAACCCCCTTTGACTGTAATTTTCCATTACCTTCCCAAATCCTATAAAACGGCCCCACCCCTATCTCCCTTCGCTGA
CTCTCTTTTCGGACTCAGCCCGCCTGCCCCCAGGTGAAATAAACAGCCATGTTGCTCACACAAAGCCTGTTTGGTGTCTC

Figure S11. KLF5 and SOX9 binding sites in *HERVH* LTRs. (**A**) KLF5 and SOX9 binding consensus sequences from the JASPAR database (https://jaspar.genereg.net). (**B**) Sequence of the 5′ and 3′ LTRs of the *HERVH Xp22*.32 (LTR7Y), *HERVH 1p31.3* (LTR7u2) and *HERVH 13q33.3* (LTR7u2), with KLF5 and SOX9 binding sites highlighted. Dark blue highlighting corresponds to a perfect KLF5 binding site (with preferred bases in all positions) and cyan highlighting corresponds to an imperfect site (with the second preferred base in one position of the KLF5 binding motif). For SOX9, no perfect binding sites were detected and red highlighting corresponds to an imperfect site (with the second preferred base in two positions of the SOX9 binding motif).

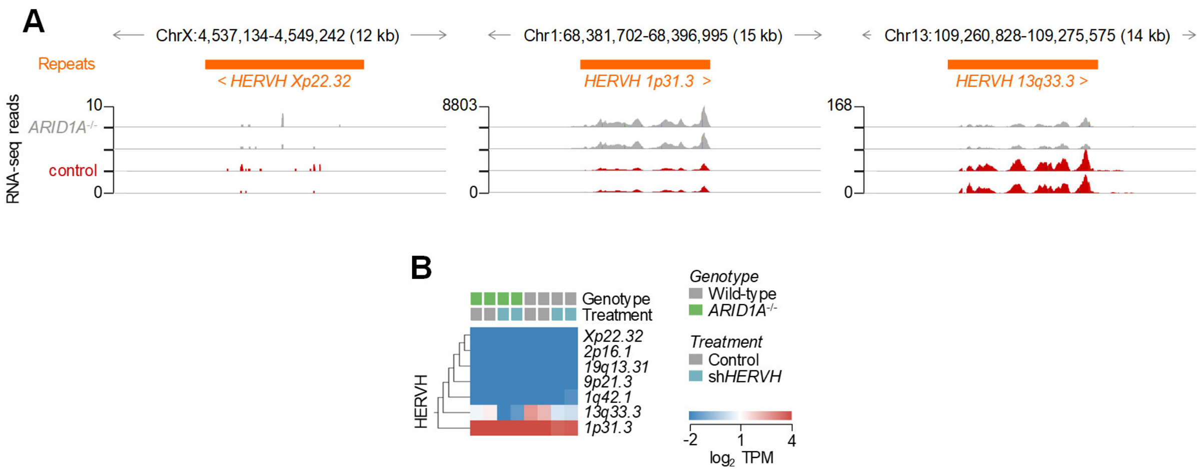

Figure S12. Regulation of individual *HERVH* proviruses by ARID1A. (**A**) RNA-seq traces of *ARID1A*$^{-/-}$ and control COAD cells HCT-116 (2 samples per genotype) at the *HERVH Xp22.*32, *HERVH 1p31.3* and *HERVH 13q33.3* proviruses, reanalyzed from GSE180475. (**B**) Heamap of expression of the indicated HERVH provirus in of *ARID1A*$^{-/-}$ and control HCT-116 cells, left untreated or treated with HERVH-targeting shRNA (sh*HERVH*), reanalyzed from GSE180475. Each column is an individual sample.

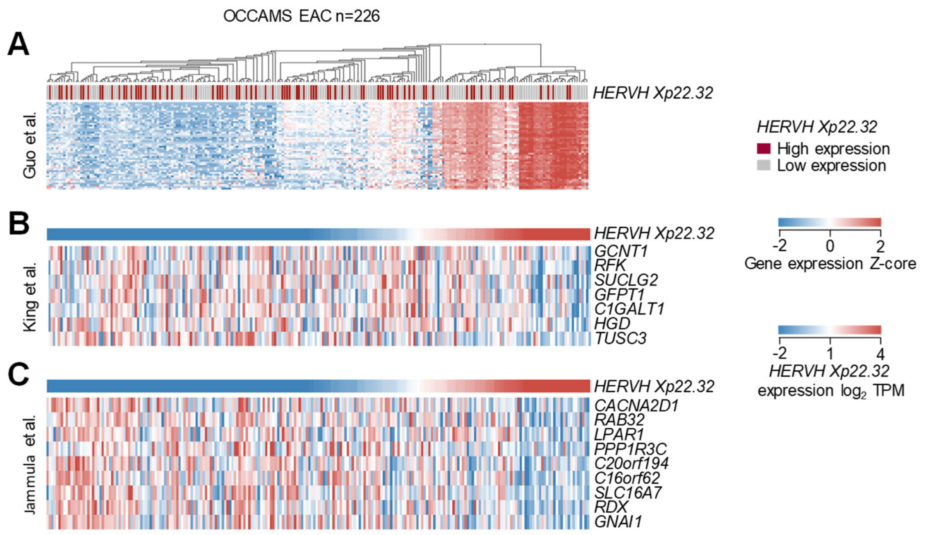

Figure S13. Lack of overlap between *HERVH Xp22.32*-defined and previously defined EAC subsets. The heatmaps show the expression in OCCAMS EAC samples (n=226) of genes that were previously reported to define molecular subtypes of EAC or ESCA. (**A**) The top 50 genes identified by Guo et al. 2018, as defining of EAC subtypes were used for hierarchical clustering of OCCAMS EAC samples. The clusters formed show no correlation with *HERVH Xp22.32* expression group (using 1 TPM as the cut-off value to define high and low *HERVH Xp22.32* expression). (**B**) The 45 genes identified King et al. 2021 as defining the metabolic differences between EAC and ESCC were tested by linear regression analyses against *HERVH Xp22.32* expression levels. The heatmap depicts the 7 of these 45 genes that correlated significantly (p=0.05, q=0.05) with *HERVH Xp22.32* expression. (**C**) The 237 genes identified Jammula et al. 2020 as having lower expression in relation to increased methylation were tested by linear regression analyses against *HERVH Xp22.32* expression levels. The heatmap depicts the 9 of these 237 genes that correlated significantly (p=0.05, q=0.05) with *HERVH Xp22.32* expression.

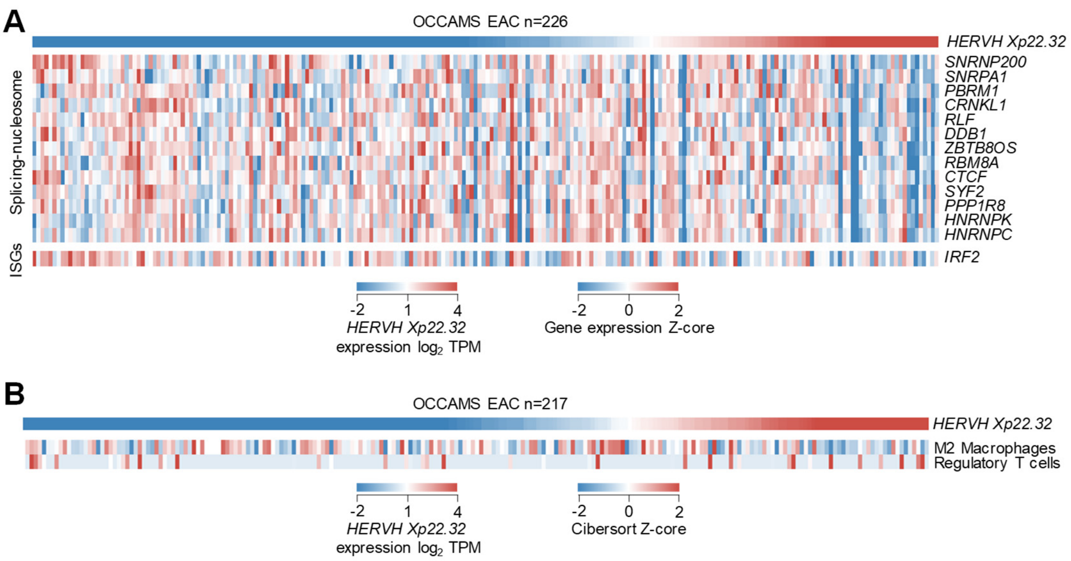

Figure S14. Transcriptional signatures associated with *HERVH Xp22.32* expression. (**A**) Heatmap of expression of selected splicing and nucleosome related genes (top) and ISGs (bottom) that correlated significantly ($p<0.05$, $q<0.05$) with *HERVH Xp22.32* expression by linear regression analyses. (**B**) Heatmap of representation (Z-scores) of the immune cell types, calculated by cellular deconvolution (Cibersort), that correlated significantly ($p<0.05$, $q<0.05$) with *HERVH Xp22.32* expression by linear regression analyses.
